# Vascular Endothelial Growth Factor C (VEGF-C) Sensitizes Lymphatic Endothelial Cells to Oxidative-Stress-Induced Apoptosis through DNA Damage and Mitochondrial Dysfunction: Implications for Lymphedema

**DOI:** 10.3390/ijms25147828

**Published:** 2024-07-17

**Authors:** Lazina Hossain, Karina Pereira Gomes, Xiaoyan Yang, Emily Liu, Jacques Du Toit, Pierre-Yves von der Weid, Spencer Bruce Gibson

**Affiliations:** 1 Department of Oncology, Faculty of Medicine and Dentistry, University of Alberta, Edmonton, AB T6G 2R3, Canada; lhossain@ualberta.ca (L.H.); kpereira@ualberta.ca (K.P.G.); xiaoyan3@ualberta.ca (X.Y.); eliu3@ualberta.ca (E.L.); dutoit@ualberta.ca (J.D.T.); 2 Department of Physiology & Pharmacology, Inflammation Research Network, Snyder Institute for Chronic Diseases, Cumming School of Medicine, University of Calgary, Calgary, AB T6G 2R3, Canada; vonderwe@ucalgary.ca

**Keywords:** lymphedema, cell death, vascular endothelial growth factor C, oxidative stress, mouse tail lymphedema

## Abstract

Secondary lymphedema is caused by damage to the lymphatic system from surgery, cancer treatment, infection, trauma, or obesity. This damage induces stresses such as oxidative stress and hypoxia in lymphatic tissue, impairing the lymphatic system. In response to damage, vascular endothelial growth factor C (VEGF-C) levels increase to induce lymphangiogenesis. Unfortunately, VEGF-C often fails to repair the lymphatic damage in lymphedema. The underlying mechanism contributing to lymphedema is not well understood. In this study, we found that surgery-induced tail lymphedema in a mouse model increased oxidative damage and cell death over 16 days. This corresponded with increased VEGF-C levels in mouse tail lymphedema tissue associated with macrophage infiltration. Similarly, in the plasma of patients with secondary lymphedema, we found a positive correlation between VEGF-C levels and redox imbalance. To determine the effect of oxidative stress in the presence or absence of VEGF-C, we found that hydrogen peroxide (H_2_O_2_) induced cell death in human dermal lymphatic endothelial cells (HDLECs), which was potentiated by VEGF-C. The cell death induced by VEGF-C and H_2_O_2_ in HDLECs was accompanied by increased reactive oxygen species (ROS) levels and a loss of mitochondrial membrane potential. Antioxidant pre-treatment rescued HDLECs from VEGF-C-induced cell death and decreased ROS under oxidative stress. As expected, VEGF-C increased the number of viable and proliferating HDLECs. However, upon H_2_O_2_ treatment, VEGF-C failed to increase either viable or proliferating cells. Since oxidative stress leads to DNA damage, we also determined whether VEGF-C treatment induces DNA damage in HDLECs undergoing oxidative stress. Indeed, DNA damage, detected in the form of gamma H2AX (γH2AX), was increased by VEGF-C under oxidative stress. The potentiation of oxidative stress damage induced by VEFG-C in HDLECs was associated with p53 activation. Finally, the inhibition of vascular endothelial growth factor receptor-3 (VEGFR-3) activation blocked VEGF-C-induced cell death following H_2_O_2_ treatment. These results indicate that VEGF-C further sensitizes lymphatic endothelial cells to oxidative stress by increasing ROS and DNA damage, potentially compromising lymphangiogenesis.

## 1. Introduction

Lymphedema is a disease of the lymphatic system that prevents the removal of lymph fluid from tissues [1]. It is a serious chronic condition characterized by swelling due to an abnormal accumulation of lymph fluid in interstitial spaces [1]. Secondary lymphedema is caused by an injury to the lymphatic system due to surgery, cancer treatment, infection, trauma, or obesity. The development of lymphedema takes place through a multi-step process involving inflammation, fibrosis, and altered layers of skin and fat cells [2]. This indicates that secondary events in addition to lymphatic insult contribute to lymphedema progression. However, there is little understanding of the mechanisms of lymphedema development.

The lymphatic microenvironment is altered in lymphedema. During lymphedema progression, lymphatic vessels become enlarged, smooth muscle coverage is reduced, adipose tissue is displaced, keratinocytes proliferate, and macrophages and Type 2 T helper cells expand [3]. Beyond cellular changes, microenvironmental stresses occur in lymphedema tissue. In lymphedema skin, hypoxia-inducible factors levels are altered and these changes exacerbate lymphatic malfunction by promoting nonproductive lymphangiogenesis [4,5]. In the plasma of patients with cancer-related lymphedema, there are increased oxidative markers, as determined by the elevated oxidized-to-reduced glutathione ratio, and lipid peroxidation products such as malondialdehyde and 4-hydroxynonenal [6]. It is, however, unclear how microenvironmental stresses contribute to lymphatic damage leading to lymphedema. 

Lymphangiogenesis occurs in response to lymphatic injury [7]. One of the key regulatory factors in lymphangiogenesis is vascular endothelial growth factor C (VEGF-C) [8]. VEGF-C binds to vascular endothelial growth factor receptor-3 (VEGFR-3), activating signaling pathways that lead to cellular proliferation, invasion, and migration [9,10]. In response to lymphatic damage, macrophage activation occurs, leading to increased levels of VEGF-C [11,12,13]. Many studies have used the delivery of VEGF-C to repair lymphatic damage in lymphedema through the induction of lymphangiogenesis; however, these studies have had limited success [14,15,16,17]. Furthermore, lymphedema patients have increased levels of VEGF-C in circulation, while mouse models of surgery-induced tail lymphedema show locally increased VEGF-C levels [18]. However, increased levels of VEGF-C fail to resolve lymphedema. Indeed, in a transgenic VEGF-C mouse model, it was demonstrated that elevated levels of VEGF-C increased tail lymphedema compared to the control mice [19]. Despite recent advances in the understanding of the pathophysiology of lymphedema, the reasons why VEGF-C fails to resolve lymphatic damage in lymphedema patients remains unclear.

In this study, we show that oxidative stress markers increase over time in a mouse tail lymphedema model, followed by increased cell death. VEGF-C levels also increased at earlier times and correlated with increased macrophage infiltration. In human dermal lymphatic endothelial cells (HDLECs), VEGF-C increases hydrogen peroxide (H_2_O_2_)-induced cell death through increased reactive oxygen species (ROS) production. VEGF-C treatment under oxidative stress also increased DNA damage in lymphatic endothelial cells (LECs). These effects were blocked by VEGFR-3 inhibition, suggesting that its activation under oxidative stress potentiates lymphedema.

## 2. Results

### 2.1. Oxidative Stress and VEGF-C Accumulate in the Mouse Tail Lymphedema Model Correlating with Cell Death

**Figure 1:**
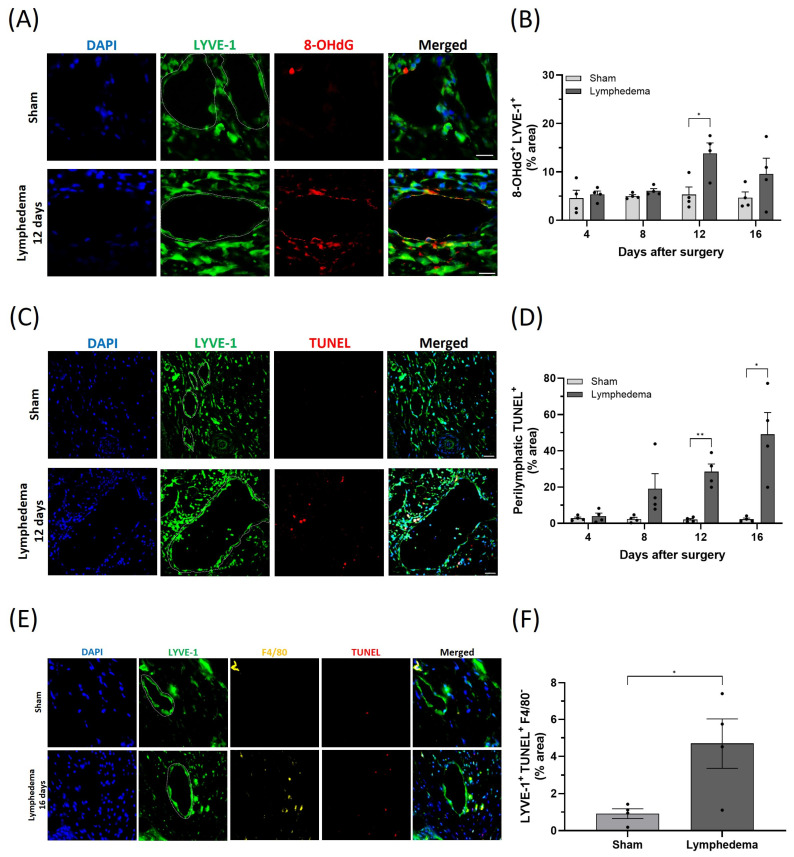
Acquired lymphedema development is associated with oxidative DNA damage in lymphatic endothelial cells (LECs) and cell death in the mouse tail model. (**A**) Representative immunofluorescence staining of LYVE-1 (green) and 8-OHdG (red) in tail tissues harvested from sham or lymphedema mice 12 days after surgery. DAPI (blue) stains the nucleus. (**B**) Quantification of 8-OHdG staining in LECs on postoperative days 4, 8, 12, and 16. (**C**) Representative immunofluorescence staining of LYVE-1 (green) and TUNEL (red) in tail tissues harvested from sham or lymphedema mice 16 days after surgery. DAPI (blue) stains the nucleus. (**D**) Quantification of TUNEL staining in the perilymphatic area on postoperative days 4, 8, 12, and 16. (**E**) Representative immunofluorescence staining of LYVE-1 (green), F4/80 (yellow), and TUNEL (red) in tail tissues harvested from sham or lymphedema mice 16 days after surgery. DAPI (blue) stains the nucleus. (**F**) Quantification of LYVE-1^+^ TUNEL^+^ F4/80^−^ staining on postoperative day 16. Scale bars: 50 μm. White-dashed lines demarcate tube-like lymphatic vessels (LYVE-1^+^). Data are presented as mean ± SEM; * *p* < 0.05, ** *p* < 0.01. DAPI: 4′,6-diamidino-2-phenylindole; LYVE-1: lymphatic vessel endothelial hyaluronan receptor 1; 8-OHdG: 8-hydroxydeoxyguanosine; TUNEL: terminal deoxynucleotidyl transferase-mediated deoxyuridine–biotin nick end labeling.

Oxidative stress in lymphedema has often been suggested, but has not been directly investigated. To determine whether oxidative damage occurs in lymphedema, lymphatic vessels in the tails of mice were surgically ablated. This lymphatic damage led to an increase in tail swelling over a 16-day period compared to sham controls (Supplementary Figure S1). At 4, 8, 12, and 16 days, mice were euthanized, and the tails were paraffin-embedded and sectioned for immunostaining. We found that 8-hydroxy-2′-deoxyguanosine (8-OHdG), a marker for oxidative DNA damage, was increased in LECs (LYVE-1^+^) 12 days after the surgery (Figure 1A,B). Using nitrotyrosine (NTYR) as marker of oxidative protein modification, we also found elevated levels of NTYR in perilymphatic tissue at earlier stages of tail lymphedema progression (Supplementary Figure S2). In addition, a terminal deoxynucleotidyl transferase dUTP nick end labeling (TUNEL) assay was performed to determine the amount of apoptosis. We found that cell death significantly increased in the perilymphatic tissue at 12 and 16 days after the lymphatic surgery (Figure 1C,D). To avoid bias against undetected perilymphatic tissue, we excluded LYVE-1^+^ macrophages using a macrophage-specific marker (F4/80) and included all remaining LYVE-1^+^ cells. We then determined the amount of apoptosis through the colocalization analysis of TUNEL- and LYVE-1-positive cells, showing that LECs underwent cell death 16 days after lymphatic injury (Figure 1E,F). When macrophages were not detected or not excluded from the analysis, we found that LYVE-1^+^ cells also showed cell death at 16 days, similar to the perilymphatic regions (Supplementary Figure S3). These data suggest that oxidative stress is increased in the mouse tail lymphedema model, followed by cell death. 

**Figure 2:**
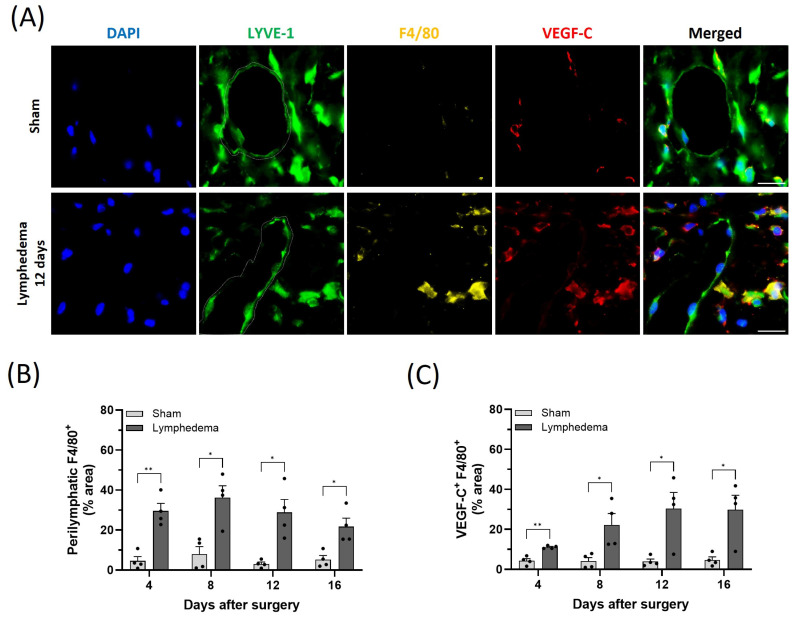
Acquired lymphedema development increases macrophage infiltration and VEGF-C in the mouse tail model. (**A**) Representative immunofluorescence staining of LYVE-1 (green), F4/80 (yellow), and VEGF-C (red) in tail tissues harvested from sham or lymphedema mice 12 days after surgery. DAPI (blue) stains the nucleus. (**B**) Quantification of F4/80 staining in the perilymphatic area on postoperative days 4, 8, 12, and 16. (**C**) Quantification of VEGF-C staining in F4/80-positive macrophages on postoperative days 4, 8, 12, and 16. Scale bars: 30 μm. White-dashed lines demarcate tube-like lymphatic vessels (LYVE-1^+^). Data are presented as mean ± SEM; * *p* < 0.05, ** *p* < 0.01. DAPI: 4′,6-diamidino-2-phenylindole; LYVE-1: lymphatic vessel endothelial hyaluronan receptor 1; VEGF-C: vascular endothelial growth factor C.

**Figure 3:**
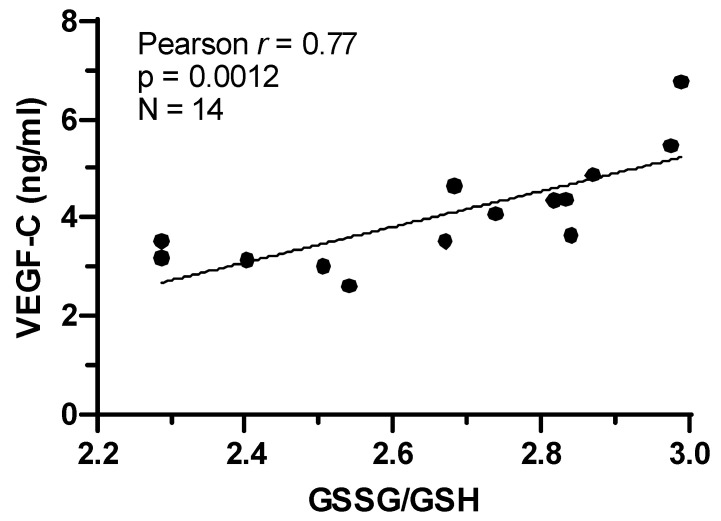
Vascular endothelial growth factor C (VEGF-C) levels are positively correlated with oxidized (GSSG)-to-reduced (GSH) glutathione ratio in plasma of patients with secondary lymphedema. Pearson correlation analysis revealed a remarkable positive correlation (r = 0.77, *p* = 0.0012) between VEGF-C concentration and redox imbalance in 14 patients with acquired lymphedema. Two-tailed paired *t*-test.

Previously, it was shown that VEGF-C is increased in mouse tail lymphedema [19]. Since the mouse tail lymphedema model showed increased oxidative damage and cell death, we determined whether VEGF-C levels were increased at the same time that oxidative damage was detected. We immunostained sham and lymphedema mouse tails for macrophages and VEGF-C. Macrophages are a natural source of VEGF-C and macrophage infiltration has previously been demonstrated in experimental lymphedema [19]. We showed that the number of macrophages was increased in the tail lymphedema model 4 days after surgery, and this elevated infiltration persisted for up to 16 days. VEGF-C levels also increased following surgery but were delayed, peaking at 12 days after surgery and remaining elevated at 16 days (Figure 2). In addition to the experimental model of acquired lymphedema, we also determined VEGF-C levels and the oxidized (GSSG)-to-reduced (GSH) glutathione ratio in the plasma of patients with secondary lymphedema. We found a significant positive correlation between increased VEGF-C levels and increased GSSG/GSH ratio (Figure 3). Thus, increased VEGF-C levels correspond with the increase in oxidative damage in both the mouse tail lymphedema model and secondary lymphedema patients. 

### 2.2. VEGF-C Sensitizes Lymphatic Endothelial Cells (LECs) to Oxidative-Stress-Induced Cell Death

**Figure 4:**
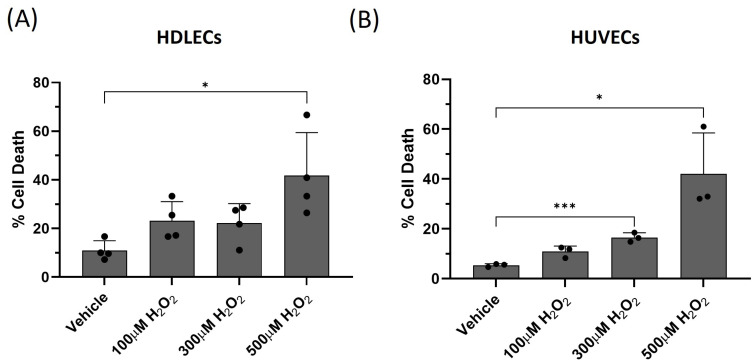
H_2_O_2_ causes significant cell death in HDLECs and HUVECs. (**A**) The bar graph represents percentage of cell death in HDLECs. HDLECs were treated with different concentrations of H_2_O_2_ for 24 h. Cell death was measured by trypan blue exclusion assay (*n* = 4). HDLECs showed significant cell death with 500 μM H_2_O_2_. (**B**) The bar graph represents percentage of cell death in HUVECs. HUVECs were treated with different concentrations of H_2_O_2_ for 24 h. Cell death was measured by trypan blue exclusion assay. Data are presented as mean ± SEM; * *p* < 0.05, *** *p* < 0.001.

To determine whether oxidative stress induces lymphatic endothelial cell death, we treated human dermal lymphatic endothelial cells (HDLECs) with hydrogen peroxide (H_2_O_2_) over a range of concentrations (100 μM to 500 µM) for 24 h. We also compared the results with human umbilical vein endothelial cells (HUVECs) to represent the effects of oxidative stress on vascular endothelial cells. In HDLECs, H_2_O_2_ at 500 μM induced 40% cell death. Similarly, H_2_O_2_ at 500 μM induced 40% cell death in HUVECs (Figure 4). H_2_O_2_ treatment also induced 50% apoptotic cell death in HDLECs, as determined by the Annexin V/7AAD assay (Supplementary Figure S4A). In addition, we compared HDLECs to rat mesenteric lymphatic endothelial cells (Rat LECs). In rat LECs, H_2_O_2_ at 500 µM induced 38% cell death (Supplementary Figure S4B). In human kidney epidermal 293 (HEK 293) cells, H_2_O_2_ treatment at 100 µM induced 75% cell death (Supplementary Figure S4C). This suggests that oxidative-stress-induced cell death occurs in LECs.

**Figure 5:**
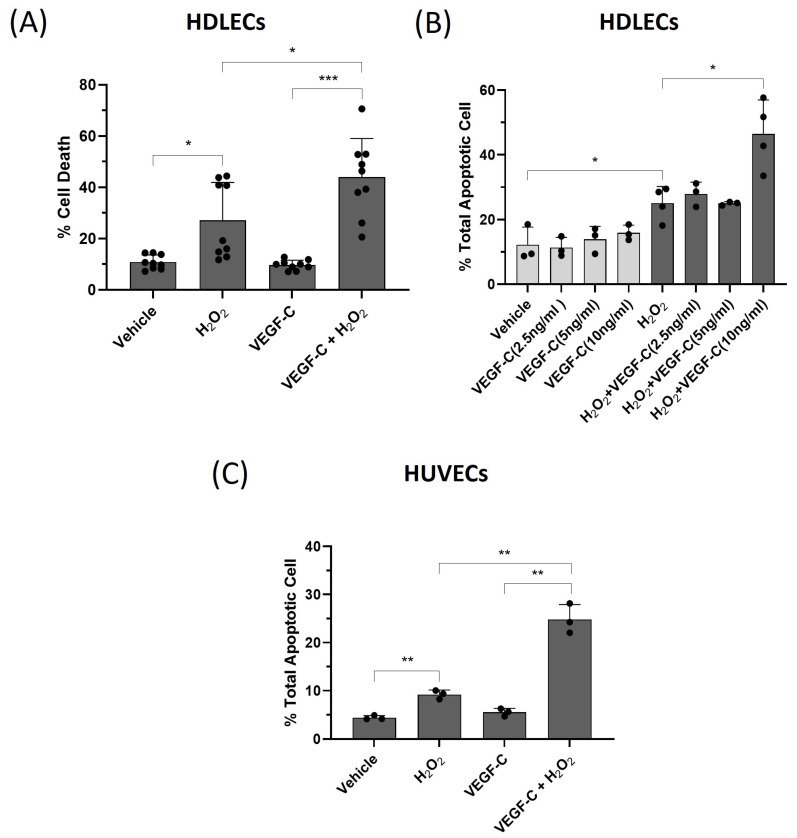
VEGF-C sensitizes HDLECs to H_2_O_2_-induced cell death through apoptosis. (**A**) The bar graph represents percentage of cell death in HDLECs. HDLECs were pretreated with 10 ng/mL of VEGF-C for 1 h before H_2_O_2_ (500 μM) treatment (*n* = 9). Cell death was measured by trypan blue exclusion assay 24 h after H_2_O_2_ treatment. (**B**) The bar graph represents the percentage of total apoptotic cell death (sum of AnnexinV^+^/7-AAD^−^ and AnnexinV^+^/7-AAD^+^) for each treatment condition (*n* = 3). HDLECs were pretreated separately with 2.5, 5, and 10 ng/mL of VEGF-C for 1 h before H_2_O_2_ (500 μM) treatment. After 24 h of treatment, HDLECs were stained with AnnexinV/7-AAD for flow cytometry assay. (**C**) The bar graph represents percentage of total apoptotic cell death in HUVECs. HUVECs were pretreated with 10 ng/mL of VEGF-C for 1 h before H_2_O_2_ (500 μM) treatment (*n* = 3). The percentage of total apoptotic cells was measured by AnnexinV/7-AAD flow cytometry assay 24 h after treatment. Data are presented as mean ± SEM; * *p* < 0.05, ** *p* < 0.01, *** *p* < 0.001.

Since H_2_O_2_ induces cell death in HDLECs and VEGF-C has been shown to be increased in lymphedema in association with the detection of oxidative stress, we determined whether VEGF-C treatment alters oxidative-stress-induced cell death in LECs. We pretreated HDLECs with VEGF-C (10 ng/mL) for 1 h and then treated the cells with 500 µM H_2_O_2_ for 24 h. Total cell death was determined by the trypan blue exclusion assay. The amount of cell death induced by H_2_O_2_ in HDLECs was 25%. However, when VEGF-C and H_2_O_2_ were combined, the amount of cell death increased to 45%, while vehicle alone or VEGF-C alone gave 10% cell death (Figure 5A). To determine whether VEGF-C sensitization to oxidative-stress-induced cell death is dose-dependent, we treated HDLECs with VEGF-C concentrations ranging from 2.5 ng/mL to 10 ng/mL alone or in combination with H_2_O_2_ (500 μM) treatment. We found that 10 ng/mL VEGF-C increased H_2_O_2_-induced cell death from 20% to 55%, as determined by the AnnexinV/7-AAD assay. No difference was found at 2.5 ng/mL or 5 ng/mL of VEGF-C (Figure 5B, Supplementary Figure S5). To confirm these results, HUVECs were treated with VEGF-C (10 ng/mL) alone or in combination with H_2_O_2_ (500 μM). We determined that H_2_O_2_ alone induced 10% cell death in HUVECs. However, cell death increased to 25% when combined with VEGF-C (Figure 5C). In addition, we treated rat LECs with VEGF-C (10 ng/mL) in combination with H_2_O_2_ (500 µM), and the amount of cell death was determined by the trypan blue assay after 24 h. Similar to HDLECs, VEGF-C in combination with H_2_O_2_ treatment increased cell death in rat LECs from 40% to 55% (Supplementary Figure S6). These data show that 10 ng/mL VEGF-C sensitizes LECs to oxidative-stress-induced cell death, and this concentration was then used for all subsequent experiments. 

VEGF-D is an important growth factor for lymphangiogenesis [10]. To investigate whether VEGF-D also sensitizes LECs to oxidative stress, HDLECs were treated with VEGF-D (10 ng/mL), followed by H_2_O_2_ treatment, as described above. We found that VEGF-D also sensitized HDLECs to H_2_O_2_-induced cell death (Supplementary Figure S7). These results indicate that both VEGF-C and VEGF-D sensitize LECs to H_2_O_2_-induced cell death.

**Figure 6:**
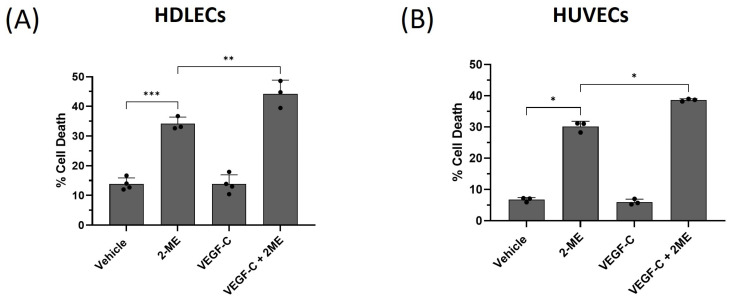
VEGF-C sensitizes HDLECs and HUVECs to 2-ME-induced cell death. The bar graphs represent percentage of cell death in HDLECs (**A**) and HUVECs (**B**), respectively. HDLECs and HUVECs were pretreated with 10 ng/mL of VEGF-C for 1 h before 2-ME (20 μM) treatment (*n* = 3). Cell death was measured by trypan blue exclusion assay 48 h after treatment. Data are presented as mean ± SEM; * *p* < 0.05, ** *p* < 0.01, *** *p* < 0.001.

An alternative method to induce oxidative stress is to incubate cells with 2-methoxyestradiol (2-ME), as this inhibits the antioxidant enzyme superoxide dismutase [20]. HDLECs were treated with 2-ME in the presence or absence of VEGF-C and the amount of cell death was determined, as described above. The combination of 2-ME and VEGF-C (10 ng/mL) significantly increased cell death from 35% to 45% in HDLECs (Figure 6A). Furthermore, HUVECs’ incubation with 2-ME and VEGF-C increased cell death from 30% to 40% after 48 h (Figure 6B). Taken together, this indicates that VEGF-C sensitizes LECs to oxidative-stress-induced cell death.

### 2.3. VEGF-C Increases Reactive Oxygen Species (ROS) in HDLECs under Oxidative Stress

**Figure 7:**
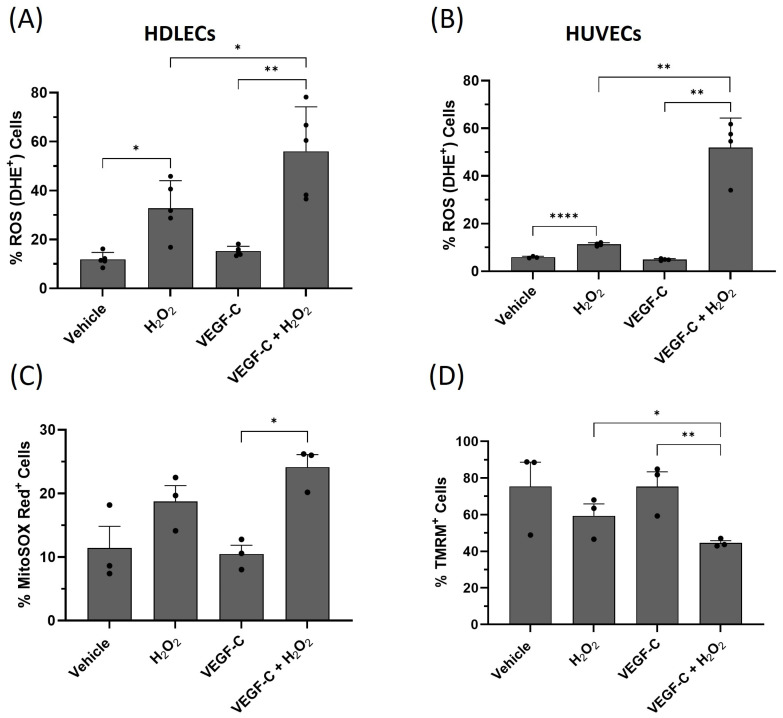
VEGF-C increases ROS levels in HDLECs and HUVECs under oxidative stress. (**A**) The bar graph represents the relative percentage of DHE (ROS)-positive HDLECs for each treatment condition (*n* = 5). (**B**) The bar graph represents the relative percentage of DHE (ROS)-positive HUVECs for each treatment condition (*n* = 4). HDLECs and HUVECs were pretreated with 10 ng/mL of VEGF-C for 1 h before H_2_O_2_ (500 μM) treatment. Flow cytometry analysis was performed to measure intracellular ROS levels in DHE-stained cells after 4 h of treatment. (**C**) The bar graph represents the percentage of MitoSOX Red-positive cells in each treatment condition. VEGF-C increased mitochondrial superoxide generation under oxidative stress after 16 h of treatment. (**D**) The bar graph represents percentage of TMRM-positive cells detected by flow cytometry. H_2_O_2_ decreases the percentage of TMRM-positive cells after 16 h of treatment compared to vehicle control. VEGF-C pretreatment further decreases the percentage of TMRM-positive cells compared to H_2_O_2_ treatment alone. Data are presented as mean ± SEM; * *p* < 0.05, ** *p* < 0.01, **** *p* < 0.0001.

To evaluate how VEGF-C is increasing H_2_O_2_-induced cell death, we measured intracellular ROS levels after H_2_O_2_ treatment. HDLECs were treated with 500 µM H_2_O_2_ for 4 h in the presence or absence of VEGF-C (10 ng/mL). Cells were then stained with dihydroethidium (DHE) to detect intracellular levels of superoxide and H_2_O_2_ by flow cytometry. As expected, treatment with H_2_O_2_ significantly increased the ROS levels from 10% to 30%. VEGF-C pretreatment further increased ROS levels under oxidative stress to 55% in HDLECs (Figure 7A, Supplementary Figure S8). Similar results were found in HUVECs (Figure 7B). Oxidative stress increased ROS through mitochondrial dysfunction. To investigate whether VEGF-C also increases mitochondrial superoxide under oxidative stress, HDLECs were stained with MitoSOX Red, which detects ROS generated in the mitochondria. The amount of mitochondrial ROS was detected by flow cytometric analysis (Figure 7C). We found that VEGF-C induced mitochondrial ROS generation under oxidative stress compared to H_2_O_2_ treatment alone. Another indicator of mitochondrial dysfunction is the loss of mitochondrial membrane potential. We measured mitochondrial membrane potential using tetramethylrhodamine-6-maleimide (TMRM). TMRM accumulates in active mitochondria with intact membrane potential. When mitochondrial membrane potential collapses in apoptotic cells, TMRM is dispersed in the cytosol and the fluorescence intensity detected by the flow cytometer is reduced. The combination of VEGF-C and H_2_O_2_ treatment reduced the TMRM fluorescence intensity in HDLECs after 16 h of treatment (Figure 7D, Supplementary Figure S9). These results indicate that VEGF-C induces ROS generation under oxidative stress.

**Figure 8:**
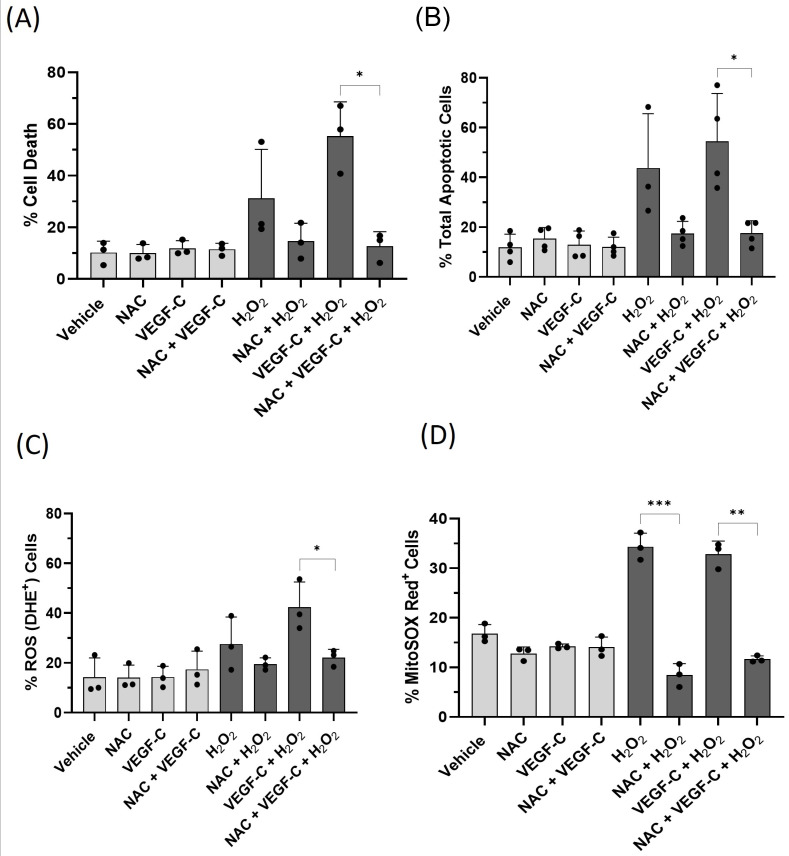
Antioxidant (NAC) treatment rescues HDLECs from VEGF-C-induced cell death by reducing intracellular ROS levels. The bar graphs represent percentage of cell death (**A**) and total apoptotic cells (**B**) in HDLECs. HDLECs were pretreated with 5 mM NAC before VEGF-C (10 ng/mL) and H_2_O_2_ (500 μM) treatment. Cell death and total apoptotic death were measured 24 h after treatment by trypan blue exclusion assay (*n* = 3) and AnnexinV/7-AAD assay (*n* = 4), respectively. Antioxidant (NAC) pre-treatment rescues HDLECs from VEGF-C-induced cell death under oxidative stress. (**C**) The bar graph represents percentage of ROS (DHE)-positive HDLECs for each treatment condition (*n* = 3). To measure the intracellular ROS level, HDLECs were stained with DHE after 4 h of treatment. NAC pre-treatment reduces intracellular ROS levels induced by VEGF-C and H_2_O_2_ treatment. (**D**) The bar graph represents percentage of MitoSOX Red-positive HDLECs in each treatment condition (*n* = 3). HDLECs were pretreated with VEGF-C (10 ng/mL) followed by H_2_O_2_ (500 μM) treatment. Mitochondrial superoxide was measured by MitoSOX Red staining 4 h after treatment. Antioxidant (NAC) pre-treatment reduces mitochondrial superoxide generation in HDLECs. Data are presented as mean ± SEM; * *p* < 0.05, ** *p* < 0.01, *** *p* < 0.001.

To determine the role of ROS in oxidative-stress-induced cell death, HDLECs were treated with 5 mM of the ROS scavenger N-Acetyl Cysteine (NAC). After NAC incubation, treatment with VEGF-C and H_2_O_2_ was performed, and the amount of cell death was measured by the trypan blue assay and AnnexinV/7-AAD assay. NAC pre-treatment prevented cell death induced by VEGF-C under oxidative stress (Figure 8A,B). In addition, NAC pre-treatment reduced intracellular and mitochondrial ROS levels in HDLECs (Figure 8C,D). These data suggest that increased ROS levels contribute to VEGF-C sensitization to oxidative-stress-induced cell death in HDLECs. 

### 2.4. VEGF-C Increases HDLEC Proliferation but Fails to Rescue Cells under Oxidative Stress

**Figure 9:**
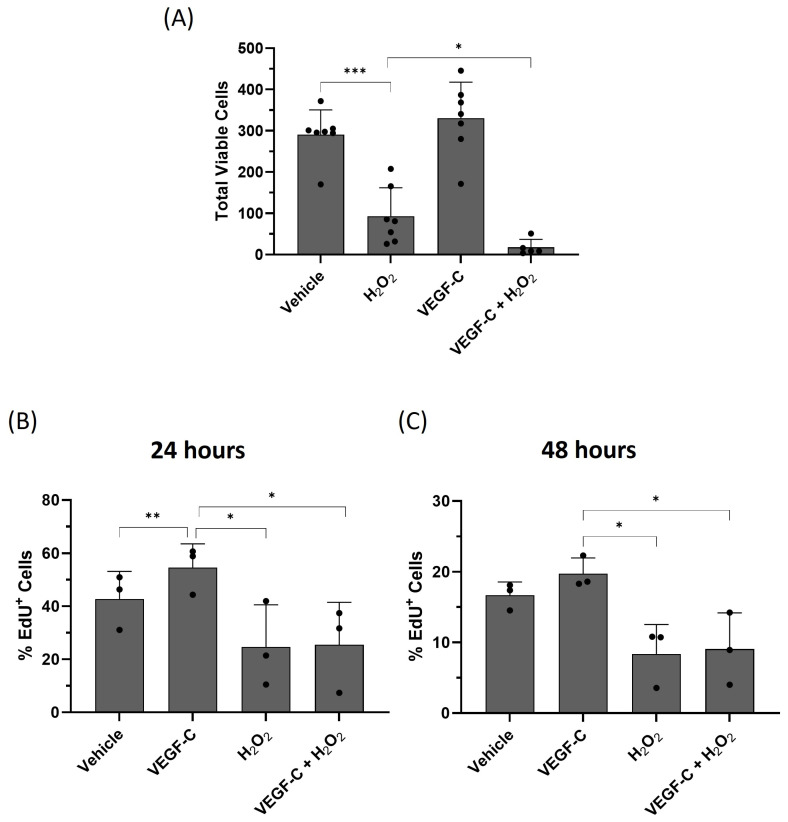
VEGF-C induces cell proliferation. (**A**) The graph represents percentage of total viable HDLECs for each treatment condition (*n* = 7). HDLECs were pretreated with 10 ng/mL of human VEGF-C 1 h before H_2_O_2_ (500 μM) treatment. After 24 h of treatment, cell culture supernatant and cells were collected and cell viability was measured by trypan blue exclusion assay. The bottom graphs represent percentage of proliferating HDLECs after 24 h (**B**) and 48 h (**C**) of treatment (*n* = 3). HDLECs were pretreated with 10 ng/mL of human VEGF-C followed by H_2_O_2_ (500 μM) treatment. After 24 h and 48 h, cells were incubated with EdU and analyzed by flow cytometry. Data are presented as mean ± SEM; * *p* < 0.05, ** *p* < 0.01, *** *p* < 0.001.

Under normal conditions, VEGF-C induces LECs [7]. To investigate the effect of oxidative stress on VEGF-C-induced HDLEC proliferation, cells were treated with 10 ng/mL of human VEGF-C 1 h before H_2_O_2_ (500 μM) treatment. After 24 h, cells were collected and resuspended in PBS to assess cell viability by the trypan blue exclusion assay. VEGF-C treatment alone increased the number of viable HDLECs, indicating an increase in cell proliferation (Figure 9A, Supplementary Figure S10). In contrast, H_2_O_2_ significantly decreased the number of viable HDLECs, which was further decreased after pre-treatment with VEGF-C. To further confirm whether VEGF-C induces HDLEC proliferation, the 5-ethynyl-2′-deoxyuridine (EdU) cell proliferation assay was performed (Figure 9B). EdU is a thymidine analog that is used to detect newly synthesized DNA during cell proliferation. The VEGF-C treatment showed increased EdU incorporation into HDLECs, but the amount of EdU-positive cells decreased with H_2_O_2_ alone and in combination with VEGF-C. This indicates that VEGF-C under normal conditions contributes to cell proliferation. However, under oxidative stress, VEGF-C fails to induce cell proliferation.

### 2.5. VEGF-C Induces DNA Damage in HDLECs under Oxidative Stress

**Figure 10:**
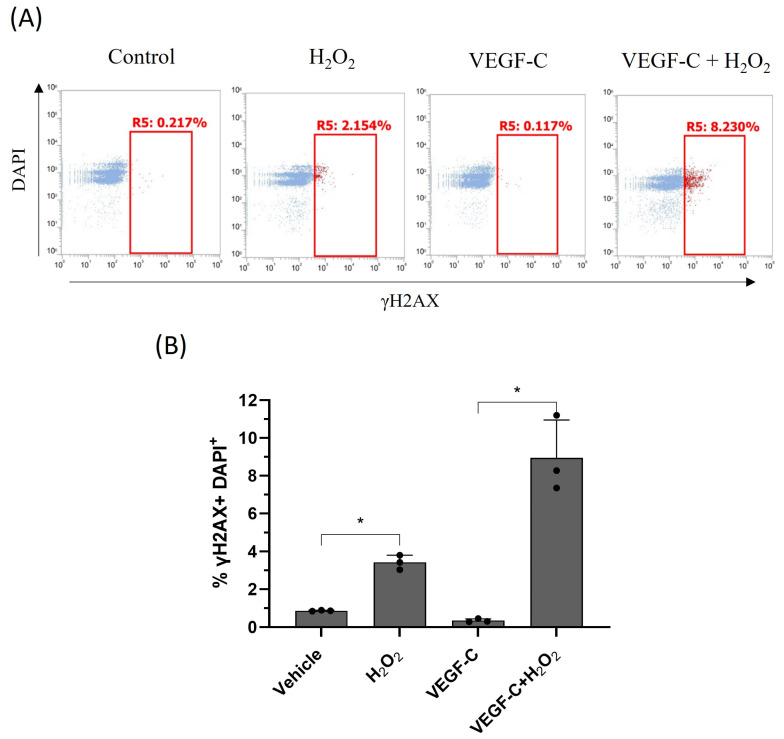
VEGF-C potentiates DNA damage in HDLECs under oxidative stress. (**A**) Representative dot plot image of γH2AX intracellular staining by flow cytometry. The X-axis represents γH2AX-positive cells and the Y-axis represents DAPI-stained cells. (**B**) Bar graph represents the percentage of γH2AX-positive stained HDLECs after 24 h of treatment with VEGF-C and H_2_O_2_ (*n* = 3). HDLECs were treated with VEGF-C and H_2_O_2_ for 24 h, as previously described, and stained with anti-γH2AX-FITC and DAPI. Data are presented as mean ± SEM; * *p* < 0.05.

Prolonged oxidative stress can cause DNA oxidation and DNA damage through apoptosis, leading to cell death [21]. To measure oxidative-stress-induced DNA damage, HDLECs were intracellularly stained with FITC-tagged γH2AX for 4 h and 24 h, following H_2_O_2_ treatment. A flow cytometric analysis was performed for γH2AX quantification. γH2AX is phosphorylated at serine 139 by DNA damage sensor protein kinase ATM in response to double-stranded DNA breaks [22]. We found that phosphorylated γH2AX levels increased in HDLECs 24 h after H_2_O_2_ treatment. We also found that VEGF-C pretreatment further increased phosphorylated γH2AX levels in HDLECs (Figure 10). These results indicate that VEGF-C allows for increased DNA damage under oxidative stress in HDLECs.

**Figure 11:**
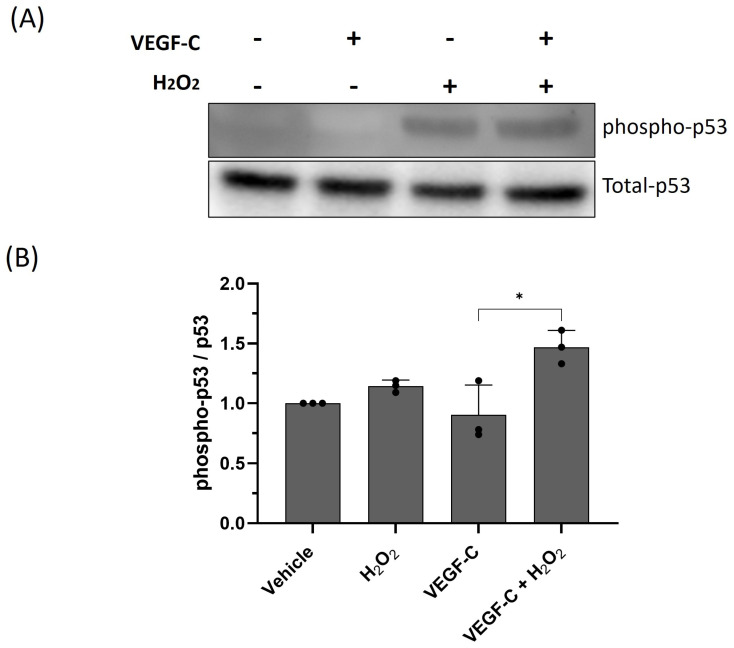
Oxidative stress activates p53. (**A**) Representative Western blot image for phosphorylated and total p53 in HDLECs 24 h after VEGF-C (10 ng/mL) and H_2_O_2_ (500 μM) treatment. (**B**) Bar graph represents the densitometry of phospho-p53 (Ser 15) normalized by total p53. Data are presented as mean ± SEM; * *p* < 0.05.

DNA damage activates p53, leading to apoptosis [23,24]. To investigate whether H_2_O_2_ and VEGF-C lead to an increased activation of p53, HDLECs treated with VEGF-C and H_2_O_2_ for 16 h were lysed and Western blotted for phosphorylated and total p53. The combination of H_2_O_2_ and VEGF-C increased p53 phosphorylation compared to each treatment alone (Figure 11). Furthermore, the p53 transcriptional target and pro-apoptotic Bcl-2 family member PUMA was also increased with the combination of H_2_O_2_ and VEGF-C (Supplementary Figure S11). Our results indicate that VEGF-C sensitizes HDLECs through increased DNA damage, leading to mitochondrial-mediated apoptosis. 

### 2.6. VEGFR-3 Inhibitor Rescued VEGF-C-Induced HDLEC Death under Oxidative Stress

In LECs, VEGF-C binds to the VEGFR-3 homodimer or VEGFR-2/3 heterodimers and activates downstream signaling pathways leading to cell proliferation [25]. To investigate the role of the VEGF-C-VEGFR-3 signaling axis in HDLECs under oxidative stress, HDLECs were treated with VEGFR-3 tyrosine kinase inhibitor (VEGFR-3i, MAZ51) in the presence or absence of VEGF-C (10 ng/mL). To confirm whether 1 µM VEGFR-3i treatment inhibited downstream signaling following VEGF-C treatment, a Western blot for phospho-ERK1/2 was performed. We showed that VEGFR-3i blocked the ERK activation induced by VEGF-C (Supplementary Figure S12).

**Figure 12:**
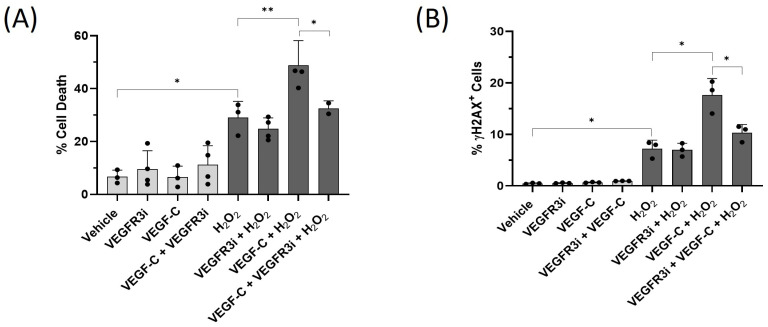
VEGFR-3 inhibitor reduces VEGF-C-induced HDLEC death and DNA damage under oxidative stress. HDLECs were pretreated with VEGFR-3 inhibitor (1 µM) for 1 h, followed by VEGF-C and H_2_O_2_ treatment, as previously described. After 24 h, cell viability was measured by trypan blue exclusion assay. DNA damage was detected by flow cytometry staining with anti-γH2AX-FITC and DAPI. (**A**) The bar graph represents the percentage of cell death measured by trypan blue exclusion assay after 24 h of treatment (*n* = 3). (**B**) The bar graph represents the percentage of γH2AX-positive stained HDLECs after 24 h of treatment (*n* = 3). Data are presented as mean ± SEM; * *p* < 0.05, ** *p* < 0.01.

We then determined whether VEGFR-3i prevented VEGF-C-sensitized HDLECs from H_2_O_2_-induced cell death. HDLECs were treated with VEGFR-3i at 1 µM in the presence or absence of VEGF-C (10 ng/mL) and H_2_O_2_ (500 µM). We found that VEGFR-3i reduces the amount of cell death induced by the VEGF-C + H_2_O_2_ combination from 50% to 35% (Figure 12A). In addition, VEGFR-3i decreases DNA damage in HDELCs after treatment with H_2_O_2_ and VEGF-C, as detected by γH2AX staining (Figure 12B, Supplementary Figure S13). This indicates that VEGF-C signaling through VEGFR-3 sensitizes LECs to oxidative-stress-induced cell death. 

## 3. Discussion

The role of oxidative stress in lymphedema remains unclear, but we have shown that oxidative damage occurs in mouse tail lymphedema and accumulates over days, followed by increased cell death. We also showed that VEGF-C levels increased, corresponding to higher oxidative stress markers. VEGF-C is a growth factor that induces proliferation in LECs under normal conditions. We showed that, under oxidative stress, VEGF-C sensitizes LECs to apoptosis. This is associated with increased DNA damage, increased ROS production, and mitochondrial dysfunction. Blocking the VEGF-C activation of VEGFR-3 reduced oxidative-stress-induced cell death and ROS levels. Our findings suggest that when LECs are exposed to oxidative stress, their activation by VEGF-C leads to apoptosis instead of inducing cellular proliferation (Supplementary Figure S14). The mechanism by which VEGF-C sensitizes lymphatic cells to oxidative-stress-induced cell death will be the focus of future investigations. 

Lymphedema is characterized by swelling in the body’s soft tissues caused by damage to the lymphatic system [1,2]. In secondary lymphedema, such damages are caused by surgeries, cancer treatments, infections, or obesity [2,15]. There are no effective treatments for lymphedema, only relief from symptoms [17]. Lymphedema progresses over time, and major changes in the microenvironment occur. There is increased inflammation associated with infiltrating T cells and macrophages. Hyperkeratosis associated with increased thickness of the skin, adipocytes surrounding lymphatic vessels, and the expansion of the diameter of lymphatic vessels are associated with increased lymphatic fluid accumulation [1,26]. At the late stages of lymphedema, fibrosis and infections also occur [26]. All these changes result in alterations in microenvironmental stresses, as demonstrated by increases in hypoxia markers in lymphedema patients’ skin samples, suggesting areas of hypoxia in lymphedema [4]. In addition, changes in microenvironmental factors occur, such as cytokine production and growth factors that contribute to oxidative stress [8]. We found that oxidative stress and VEGF-C might contribute to lymphedema progression by sensitizing LECs to apoptosis, and thus prevent lymphatic repair.

Increased oxidative markers in the plasma of breast-cancer-related lymphedema patients indicate increased oxidative stress in lymphedema [6]. This agrees with our findings that VEGF-C level corelates with oxidative stress. In addition, treating mice with antioxidants reduced surgery-induced tail lymphedema, suggesting that oxidative stress plays an important role in the progression of lymphedema [27,28]. In patients with chronic lymphedema, sodium selenite treatment decreased ROS levels in plasma and reduced lymphedema swelling [29,30]. In contrast, cancer-related lymphedema patients treated with antioxidants failed to demonstrate reduced swelling [31]. This could be due to oxidative damage having already occurred. Another way to reduce lymphedema burden is lymphatic bypass surgery, such as lymphaticovenous anastomosis (LVA). It was found that LVA reduced oxidative markers and antioxidant enzyme capacity in lymphedema patients, suggesting that lymphedema burden is associated with oxidative stress [32]. This correlates with our data that oxidative stress increases over time in mouse tail lymphedema. The mouse tail model is a popular model used to study lymphedema due to its simplicity and reproducibility. A particular advantage of the mouse tail model is that the gradual histopathological changes allowed us to study the progression of the disease from injury to the chronic phase. However, a translational limitation of the mouse tail lymphedema model is its rapid spontaneous resolution [33,34]. To address this, in our future studies, we will evaluate lymphedema progression using the irradiation-supplemented mouse hindlimb model that more closely reflects chronic human lymphedema. 

VEGF-C is a key growth factor in the proliferation of LECs during lymphangiogenesis [25]. Indeed, lymphedema patients have increased VEGF-C levels [25,35]. In addition, surgically induced tail lymphedema in mice shows increased VEGF-C levels, mediated by activated macrophages infiltrating the lymphatic tissue [19,36,37,38]. This was corroborated by our study. The overexpression of VEGF-C in a transgenic mouse model led to aggravated lymphedema with increased immune cell infiltration and vascular leakage, compared with wild-type mice [19]. Conversely, the blockage of VEGF-C by the overexpression of soluble VEGFR-3 attenuated lymphedema development, diminishing inflammation and vascular leakage [19]. In contrast, lentiviral overexpressing VEGF-C in mesenchymal stem cells (MSCs) injected in post-surgery-induced tail lymphedema mice showed increased lymphatic contraction frequency and reduced tail swelling [14]. This has been the rationale for VEGF-C-based therapies for lymphedema patients. Indeed, MSCs inhibit and ameliorate lymphedema through their antioxidant properties, which might mitigate the effects of VEGF-C on oxidative-induced cell death [39]. Unfortunately, this has resulted in limited success in reducing lymphedema in patients [17]. Our results indicate that microenvironmental stresses such as oxidative stress need to be taken into consideration in this context. VEGF-C-based therapies could be successful in microenvironments where oxidative stress is reduced or absent, but may fail in microenvironments where oxidative stress is elevated. 

## 4. Materials and Methods

### 4.1. Animals

All procedures involving animals were in compliance with the University of Alberta institutional guidelines, which conform to the guidelines published by the Canadian Council on Animal Care. Adult (8- to 12-week-old) female C57BL/6 mice were housed in standard animal cages and maintained in a constant environment with controlled room temperature, humidity, and light–dark cycle. Mice had access to laboratory chow pellets and drinking water ad libitum throughout the study. The 36 mice used in this study were purchased from Charles River Laboratories and kept at the animal facilities of the Health Sciences Laboratory Animal Services of the University of Alberta. All the experiments were approved by the Animal Care and Use Committee of the University of Alberta (AUP4047).

### 4.2. Mouse Tail Model of Secondary Lymphedema

To evaluate lymphedema, secondary lymphedema was surgically induced in the tails of the mice through the ablation of lymphatic trunks (lymphatic surgery), as previously described [4,14,18,27,28,34,37]. Briefly, mice were anesthetized with 2–3% isoflurane and kept on a heating pad, with body temperature maintained at 36.5–37.5 °C. A 3 mm full-thickness skin circumferential incision was made 2 cm distal to the base of the mouse tail. Lymphatic trunks were ablated using microsurgical scissors under a surgical microscope. Mice of the sham surgery group received skin incision only, while the primary collecting lymphatics were left intact. All mice that showed any signs of tail necrosis or infection after the surgical procedure were excluded from the study. Tail circumference measurements were taken daily with a digital caliper at every 5 mm, starting at the surgical site and going distally towards the tip of the tail. The mouse tail volume was calculated by the truncated cone equation. To characterize the progression of lymphedema from the injury to the chronic phase, mice were euthanized 4, 8, 12, and 16 days after surgery, and the tails were collected for further analysis.

### 4.3. Histology and Immunofluorescence

To determine oxidative markers in lymphedema, tissues were fixed overnight in 10% formalin (Thermo Fisher Scientific, Waltham, MA, USA), decalcified using 10% ethylenediaminetetraacetic acid (EDTA; Sigma-Aldrich, St. Louis, MO, USA), and subsequently embedded in paraffin. Hematoxylin and eosin (H&E) or immunofluorescence (IF) staining was performed using 5 µm sections according to standard techniques. For IF staining, the rehydrated sections underwent heat-mediated antigen unmasking with sodium citrate (Thermo Fisher Scientific), and subsequently overnight incubation at 4 °C with Anti-LYVE-1 (1:200; catalog PA1-16636, Thermo Fisher Scientific), Anti-8-OHdG (1:100; catalog ab48508, Abcam, Cambridge, UK), Anti-F4/80 (1:200, catalog MA1-91124, Thermo Fisher Scientific), Anti-NTYR (1:100; catalog ab125106, Abcam), and/or Anti-VEGF-C (1:100, catalog MA5-37500, Thermo Fisher Scientific). Secondary antibodies were labeled with the fluorochromes Alexa Fluor 488, Alexa Fluor 555, or Alexa Fluor 647 (1:400; Thermo Fisher Scientific). Nuclei were stained with 4′,6-diamidino-2-phenylindole (DAPI) (Sigma-Aldrich). H&E and IF slides were evaluated using a Optika B-290TB (Optika, Ponteranica, Italy) brightfield microscope and a ZEISS Axio Imager 2 (ZEISS, Oberkochen, Germany) fluorescent microscope, respectively. Fluorescence area and colocalization analyses were performed using Fiji software version 1.54f (National Institutes of Health, Bethesda, MD, USA) with a minimum of 5 high-powered fields per animal by two blinded reviewers. To quantify perilymphatic cells, positive cells were identified within a 100 µm radius of LYVE-1^+^ lymphatic vessels. For the colocalization analysis with LYVE-1^+^ lymphatics, vessel regions of interest were drawn after LYVE-1^+^ staining and morphological identification (Supplementary Figure S3). Nuclear colocalization analyses were normalized by DAPI nuclear staining.

### 4.4. TUNEL Assay

To determine the amount of cell death in lymphedema tissue, the TUNEL method was performed to label apoptotic DNA fragmentation in the tail sections. The TUNEL assay was carried out with the Click-iTTM Plus TUNEL Apoptosis Assay Kit (catalog C10618, Thermo Fisher Scientific) combined with Anti-LYVE-1, according to the manufacturer’s instruction. The images of TUNEL-positive cells were captured using a ZEISS Axio Imager 2 fluorescent microscope and subsequently quantified using Fiji software.

### 4.5. Human Study Population and Blood Sampling

To study lymphedema in humans, human subjects were recruited and blood samples collected. These procedures were approved by the Ethics Committee of the University of Alberta (Pro00115131). Each subject provided written informed consent. Anticoagulated venous blood samples were collected from 14 patients (13 women, 1 men) with secondary lymphedema following cancer treatment, injury, or chronic venous insufficiency. The mean age of the patients was 66 ± 3.7 years (mean ± SEM). Samples were centrifuged at 1500× *g* for 15 min at 4 °C, and plasma sample aliquots were stored at −80 °C for subsequent analysis.

### 4.6. VEGF-C and Glutathione Measurements

To correlate VEGF-C levels with oxidative stress in the blood samples, the VEGF-C Human ELISA Kit (catalog BMS297-2, Thermo Fisher Scientific) was used to determine the plasma levels of VEGF-C, according to the manufacturer’s instructions. This assay employs the quantitative sandwich enzyme immunoassay technique. To determine oxidative stress levels, the Glutathione Colorimetric Detection Kit (catalog EIAGSHC, Thermo Fisher Scientific) was used to quantitatively measure total, reduced (GSH), and oxidized glutathione (GSSG) in the plasma samples, according to the manufacturer’s instructions. The GSSG-to-GSH ratios were subsequently computed as an indicator of oxidative stress.

### 4.7. Cell Lines

Three different cell lines were used in this study. Human dermal lymphatic endothelial cells (HDLECs) (C-12217) were purchased from PromoCell (Heidelberg, Germany), human umbilical vein endothelial cells (HUVECs) (C0035C) were purchased from Thermo Fisher Scientific (Waltham, MA, USA), and rat mesenteric lymphatic endothelial cells (ratLECs) were kindly provided by Dr. Pierre-Yves von der Weid from the University of Calgary. All cells were maintained in endothelial cell growth medium-2 with supplements (C-22121, PromoCell) in a humidified incubator at 5% CO_2_ and 37 °C, and were used at passages 5 to 8.

### 4.8. Reagents and Antibodies for In Vitro Experiments

Hydrogen peroxide (H_2_O_2_) (H325-500) was purchased from Fisher Scientific (Ottawa, ON, Canada). Human VEGF-C (SRP3184), VEGFR-3 kinase inhibitor (MAZ51, 6764920), sodium orthovanadate (Na3VO4, 450243), and trypan blue solution (T8154) were purchased from Sigma-Aldrich (St. Louis, MO, USA). The antioxidants 2-methoxyestradiol (2-ME, HY-12033) and N-acetyl-L-Cysteine (NAC, 20216-10) were purchased from Cedarlane Labs (Burlington, ON, Canada). Protease and phosphatase inhibitor cocktails (100×) (78440) were purchased from Thermo Fisher Scientific (Rockford, IL, USA). The following primary antibodies were used for the Western blot: p53 (DO-1) (sc-126, Santa Cruz Biotechnology); phopho-p53 (Ser 15) (9284, Cell Signaling Technology, Danvers, MA, USA); total ERK (1/2) (4696, Cell Signaling Technology); and phosphor-ERK (1/2) (4370, Cell Signaling Technology). Secondary antibodies of HRP goat anti-mouse IgG (926-80010) and HRP goat anti-rabbit IgG (926-80011) were purchased from LI-COR Biotechnology (Lincoln, NE, USA).

### 4.9. Trypan Blue Exclusion Assay

To determine levels of cell death, HDLECs (80,000–100,000 cells), HUVECs (80,000–100,000 cells), and ratLECs (35,000–45,000 cells) were seeded into 60 mm Petri dishes (Sarstedt, Montreal, QC, Canada). The cells were allowed to grow for 24 h–48 h before treatment. On the day of treatment, fresh media were added before the addition of inhibitors or drugs. If inhibitors were tested, cells were pretreated with inhibitors for 1 h; then, the drugs were added and incubated at 37 °C for 24 h–48 h, unless otherwise indicated. DMSO or PBS was added as the vehicle control. At the end of the treatment, cells were collected with the media, resuspended in PBS, and stained with 0.04% trypan blue (Sigma, Oakville, ON, Canada) at a ratio of 1:1. Live and dead cells were counted using a hemocytometer. Total cell death was calculated as follows: total cell death (%) = dead cells/(live cells + dead cells) × 100.

### 4.10. Annexin V/7AAD Assay

After the treatment with VEGF-C and H_2_O_2_ for 24h, HDLECs and HUVECs were collected and stained with annexin V and 7-AAD (BD Biosciences, San Jose, CA, USA), following the manufacturer’s protocol to determine the amount of apoptosis. Data were acquired using the Attune NxT flow cytometer (Life Technology, Thermo Fisher Co). A total of 10,000 events were collected and gated using FITC (annexin V) and BL1 (7-AAD) channels. Events with annexin V-positive results were considered apoptotic cells. Data were analyzed using FlowJo version 10.10 (Tree Star Inc., Ashland, OR, USA) analysis software.

### 4.11. ROS Detection

To evaluate the ROS levels in lymphatic cells, cells were seeded in 60 mm Petri dishes and treated with VEGF-C, H_2_O_2_, and/or NAC. After 4 h or 24 h of treatment, cells were collected with the media, resuspended in PBS, and stained with 3.2 µM dihydroethidium (DHE, Invitrogen) for 30 min at 37 °C. Mitochondrial superoxide was detected by staining the treated cells with 1 μM of MitoSox Red (M36008, ThermoFisher) for 30 min at 37 °C. ROS levels were determined using an Attune NxT flow cytometer, and data were analyzed using FlowJo software version 10.10. A total of 10,000 events were collected for each sample.

### 4.12. Mitochondrial Membrane Potential Detection

To determine the amount of mitochondrial dysfunction, HDLECs were seeded in 60mm Petri dishes and treated with VEGF-C and H_2_O_2_. After 16 h, cells were collected with the media, resuspended in PBS, and incubated with 100 nM Tetramethylrhodamine (TMRM, T668, ThermoFisher) for 30 min at 37 °C. Cells were analyzed using an Attune NxT flow cytometer with a 488 nm/570 nm filter. A total of 10,000 events were collected for data analysis with FlowJo software version 10.10. This was used to determine the mitochondrial membrane potential in the cells. 

### 4.13. EDU Proliferation Assay

Cell proliferation was determined using the EdU staining proliferation kit (ab219801, Abcam) following the manufacturer’s protocol. Briefly, after 24 h and 48 h of treatment, cells were stained with EdU solution and incubated for 2 h at 37 °C. Cells were then harvested and fixed with 4% formaldehyde for 15 min at room temperature, followed by permeabilization for 15 min. Fluorescent signals were developed by incubation with the EdU reaction mix for 30 min, and analyzed with an Attune NxT flow cytometer. FlowJo software version 10.10 was used for data analysis.

### 4.14. γH2AX Detection

To detect DNA damage, HDLECs were seeded in 60 mm Petri dishes and treated, as indicated. After 24 h of treatment, cells were harvested, fixed, and permeabilized using a transcription factor buffer set (562574, BD Bioscience) according to the manufacturer’s protocol. Cells were then incubated with Alexa 488-tagged γH2AX (05-636-AF488, MilliporeSigma, Burlington, MA, USA) for 1h and washed twice before DAPI staining. An Attune NxT flow cytometer and FlowJo software version 10.10 were used for analysis.

### 4.15. Western Blotting

To evaluate protein expression, protein lysates were prepared in NP40 lysis buffer supplemented with protease and phosphatase inhibitors. Protein concentrations were determined by the Pierce BCA Protein Assay kit (23225, Thermo Scientific) and measured using a FLUOstar Omega multi-plate reader (BMG Labtech, Ortenberg, Germany). Amounts of 20–30 µg of total protein were loaded, separated with Tris-glycine sodium dodecylsulfate polyacrylamide gel electrophoresis, and transferred onto nitrocellulose membranes using a Trans-Blot Turbo (Bio-Rad, Hercules, USA). Primary antibodies were diluted in 0.1% TBS-T and incubated at 4 °C overnight followed by 1 h incubation with HRP-conjugated secondary antibodies. After being developed by clarity Western ECL substrate (170-5060, Bio-Rad), the membranes were imaged using the UVP ChemStudio gel imaging system (Analytikjena, Upland, CA, USA), and quantified using VisionWorks software version 8.20 (Analytikjena).

### 4.16. Statistical Analysis

All graphs and statistical analyses were generated using GraphPad Prism version 10.2.3. Pearson correlation analysis was used to determine the correlations between human plasma analytes. The significance was determined by paired *t*-tests. For the mouse data, differences between groups were identified using two-way ANOVA followed by Šídák’s post hoc test. For the in vitro data, statistical significance was determined using one-way ANOVA followed Tukey’s post hoc test or an unpaired *t*-test. Data are presented as mean ± standard error of the mean (SEM) and p values lower than 0.05 were considered statistically significant.

## 5. Conclusions

This study gives new insight into how VEGF-C sensitizes LECs to apoptosis under oxidative stress through increased DNA damage and ROS. This might explain why VEGF-C levels are increased in lymphedema without reducing the disease burden. In addition, it gives context as to why VEGF-C treatments are currently ineffective for lymphedema where oxidative stress conditions are expected. Thus, it is important to understand the relationship between microenvironmental stresses and growth factors, such as VEGF-C, to improve treatment strategies for lymphedema.

## Data Availability

Data are contained within the article and Supplementary Materials.

## Supplementary Materials

The following supporting information can be downloaded at: https://www.mdpi.com/article/10.3390/ijms25147828/s1.
