# Supplementary material for: Vascular Endothelial Growth Factor C (VEGF-C) Sensitizes Lymphatic Endothelial Cells to Oxidative-Stress-Induced Apoptosis through DNA Damage and Mitochondrial Dysfunction: Implications for Lymphedema
Source: Int J Mol Sci. 2024 Jul 17;25(14):7828. doi: 10.3390/ijms25147828 (PMC11277328; doi:10.3390/ijms25147828)
Supplement: Supplementary file 1 [file ijms-25-07828-s001.zip › ijms-3035085-supplementary.pptx]

## Slide 1
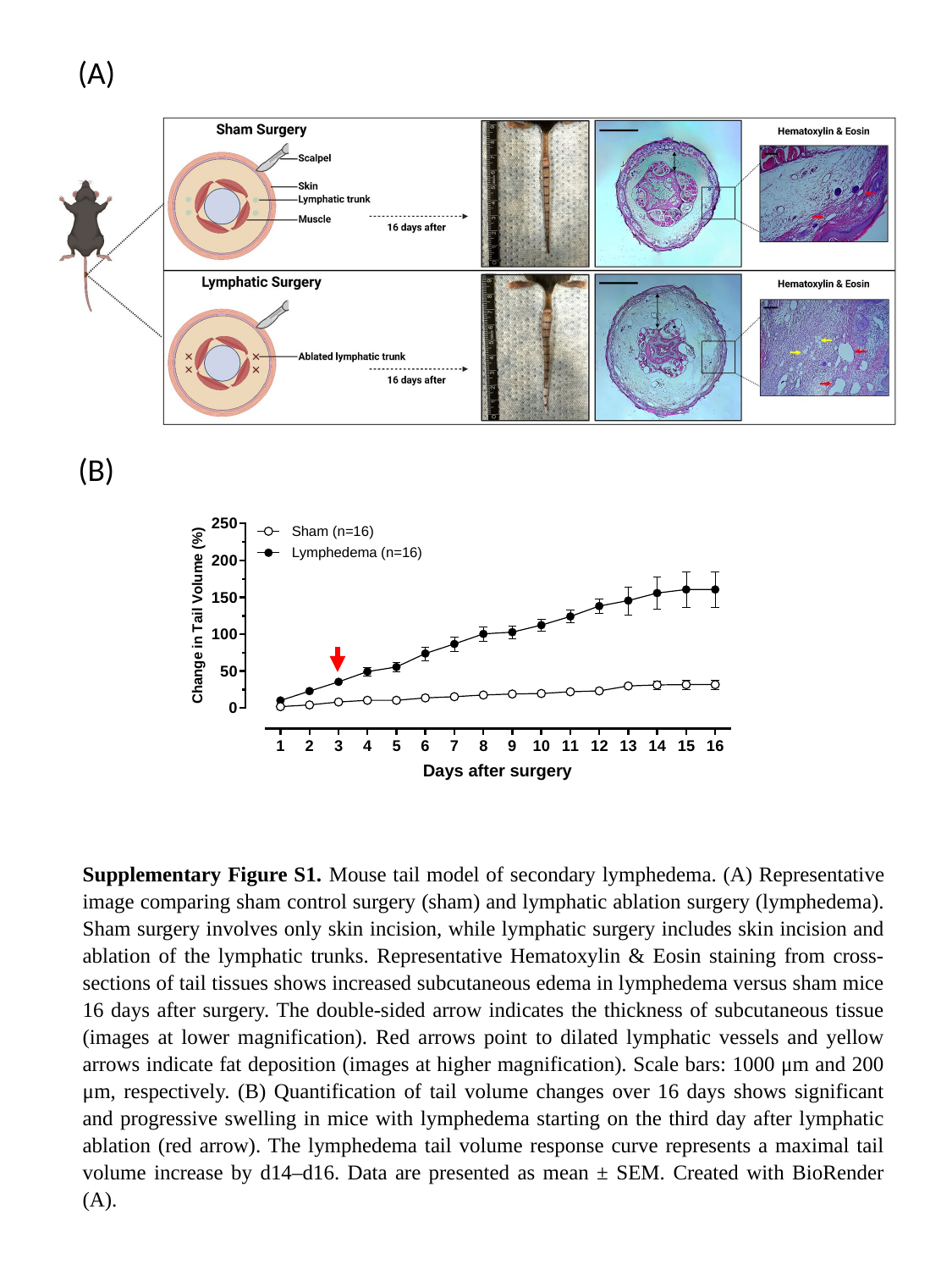

(A)
(B)
Supplementary Figure S1. Mouse tail model of secondary lymphedema. (A) Representative image comparing sham control surgery (sham) and lymphatic ablation surgery (lymphedema). Sham surgery involves only skin incision, while lymphatic surgery includes skin incision and ablation of the lymphatic trunks. Representative Hematoxylin & Eosin staining from cross-sections of tail tissues shows increased subcutaneous edema in lymphedema versus sham mice 16 days after surgery. The double-sided arrow indicates the thickness of subcutaneous tissue (images at lower magnification). Red arrows point to dilated lymphatic vessels and yellow arrows indicate fat deposition (images at higher magnification). Scale bars: 1000 μm and 200 μm, respectively. (B) Quantification of tail volume changes over 16 days shows significant and progressive swelling in mice with lymphedema starting on the third day after lymphatic ablation (red arrow). The lymphedema tail volume response curve represents a maximal tail volume increase by d14–d16. Data are presented as mean ± SEM. Created with BioRender (A).

## Slide 2
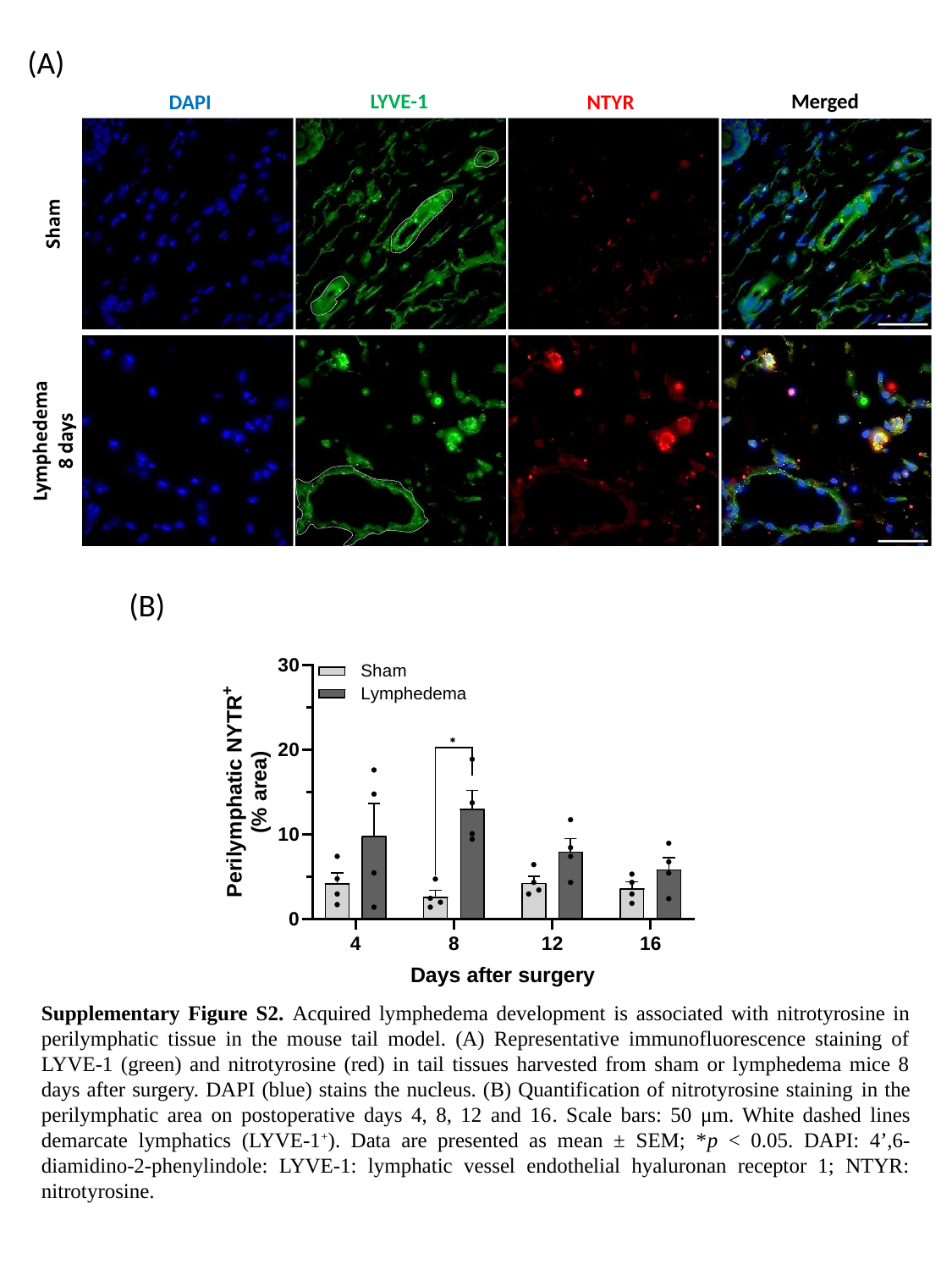

(A)
LYVE-1
Merged
DAPI
NTYR
(B)
Supplementary Figure S2. Acquired lymphedema development is associated with nitrotyrosine in perilymphatic tissue in the mouse tail model. (A) Representative immunofluorescence staining of LYVE-1 (green) and nitrotyrosine (red) in tail tissues harvested from sham or lymphedema mice 8 days after surgery. DAPI (blue) stains the nucleus. (B) Quantification of nitrotyrosine staining in the perilymphatic area on postoperative days 4, 8, 12 and 16. Scale bars: 50 μm. White dashed lines demarcate lymphatics (LYVE-1+). Data are presented as mean ± SEM; *p < 0.05. DAPI: 4’,6-diamidino-2-phenylindole: LYVE-1: lymphatic vessel endothelial hyaluronan receptor 1; NTYR: nitrotyrosine.

## Slide 3
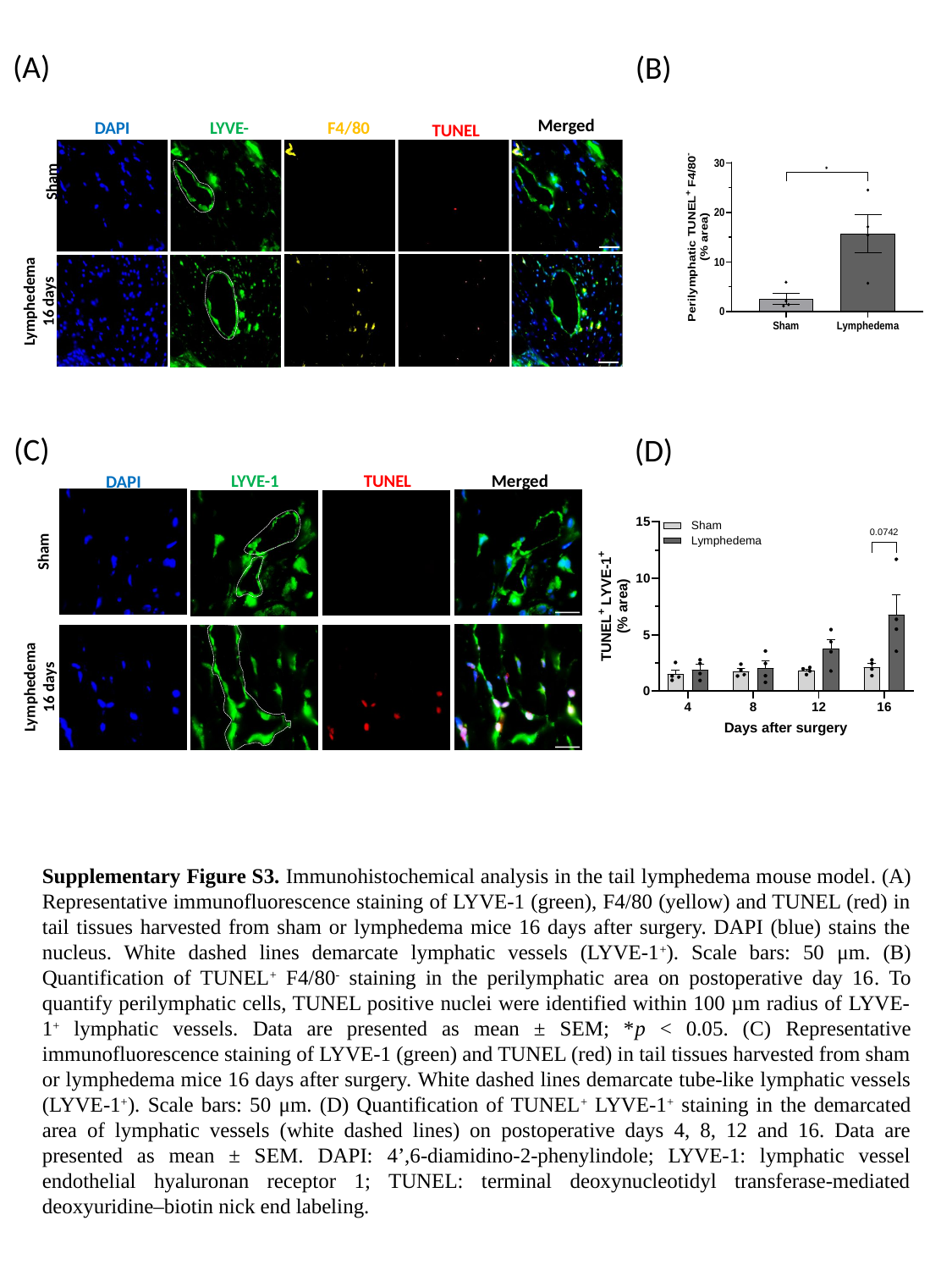

(A)
(B)
Merged
DAPI
LYVE-1
F4/80
TUNEL
Sham
Lymphedema
16 days
(C)
(D)
LYVE-1
TUNEL
Merged
DAPI
Sham
Lymphedema
16 days
Supplementary Figure S3. Immunohistochemical analysis in the tail lymphedema mouse model. (A) Representative immunofluorescence staining of LYVE-1 (green), F4/80 (yellow) and TUNEL (red) in tail tissues harvested from sham or lymphedema mice 16 days after surgery. DAPI (blue) stains the nucleus. White dashed lines demarcate lymphatic vessels (LYVE-1+). Scale bars: 50 μm. (B) Quantification of TUNEL+ F4/80- staining in the perilymphatic area on postoperative day 16. To quantify perilymphatic cells, TUNEL positive nuclei were identified within 100 µm radius of LYVE-1+ lymphatic vessels. Data are presented as mean ± SEM; *p < 0.05. (C) Representative immunofluorescence staining of LYVE-1 (green) and TUNEL (red) in tail tissues harvested from sham or lymphedema mice 16 days after surgery. White dashed lines demarcate tube-like lymphatic vessels (LYVE-1+). Scale bars: 50 μm. (D) Quantification of TUNEL+ LYVE-1+ staining in the demarcated area of lymphatic vessels (white dashed lines) on postoperative days 4, 8, 12 and 16. Data are presented as mean ± SEM. DAPI: 4’,6-diamidino-2-phenylindole; LYVE-1: lymphatic vessel endothelial hyaluronan receptor 1; TUNEL: terminal deoxynucleotidyl transferase-mediated deoxyuridine–biotin nick end labeling.

## Slide 4
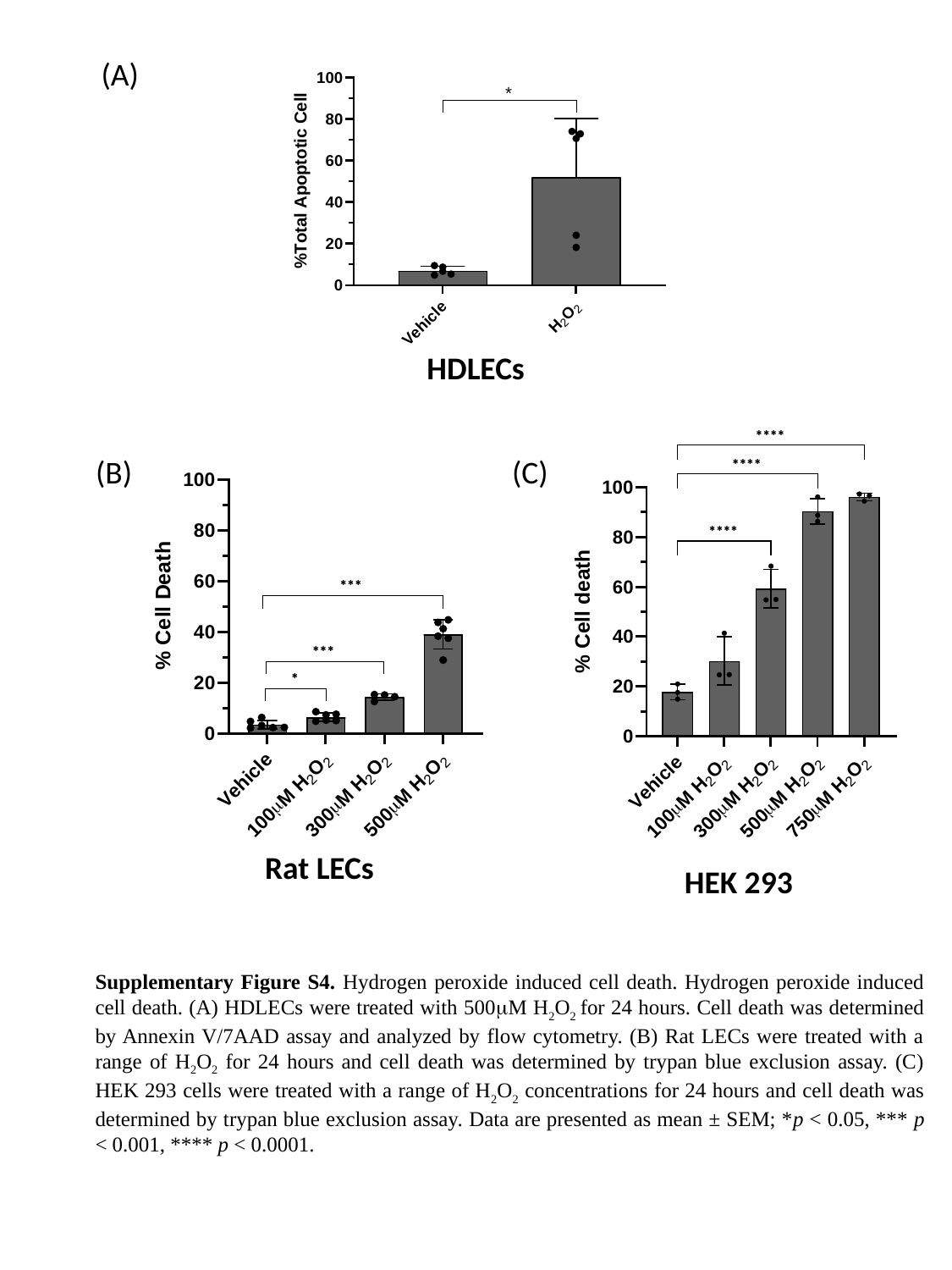

(A)
HDLECs
(C)
(B)
Rat LECs
HEK 293
Supplementary Figure S4. Hydrogen peroxide induced cell death. Hydrogen peroxide induced cell death. (A) HDLECs were treated with 500mM H2O2 for 24 hours. Cell death was determined by Annexin V/7AAD assay and analyzed by flow cytometry. (B) Rat LECs were treated with a range of H2O2 for 24 hours and cell death was determined by trypan blue exclusion assay. (C) HEK 293 cells were treated with a range of H2O2 concentrations for 24 hours and cell death was determined by trypan blue exclusion assay. Data are presented as mean ± SEM; *p < 0.05, *** p < 0.001, **** p < 0.0001.

## Slide 5
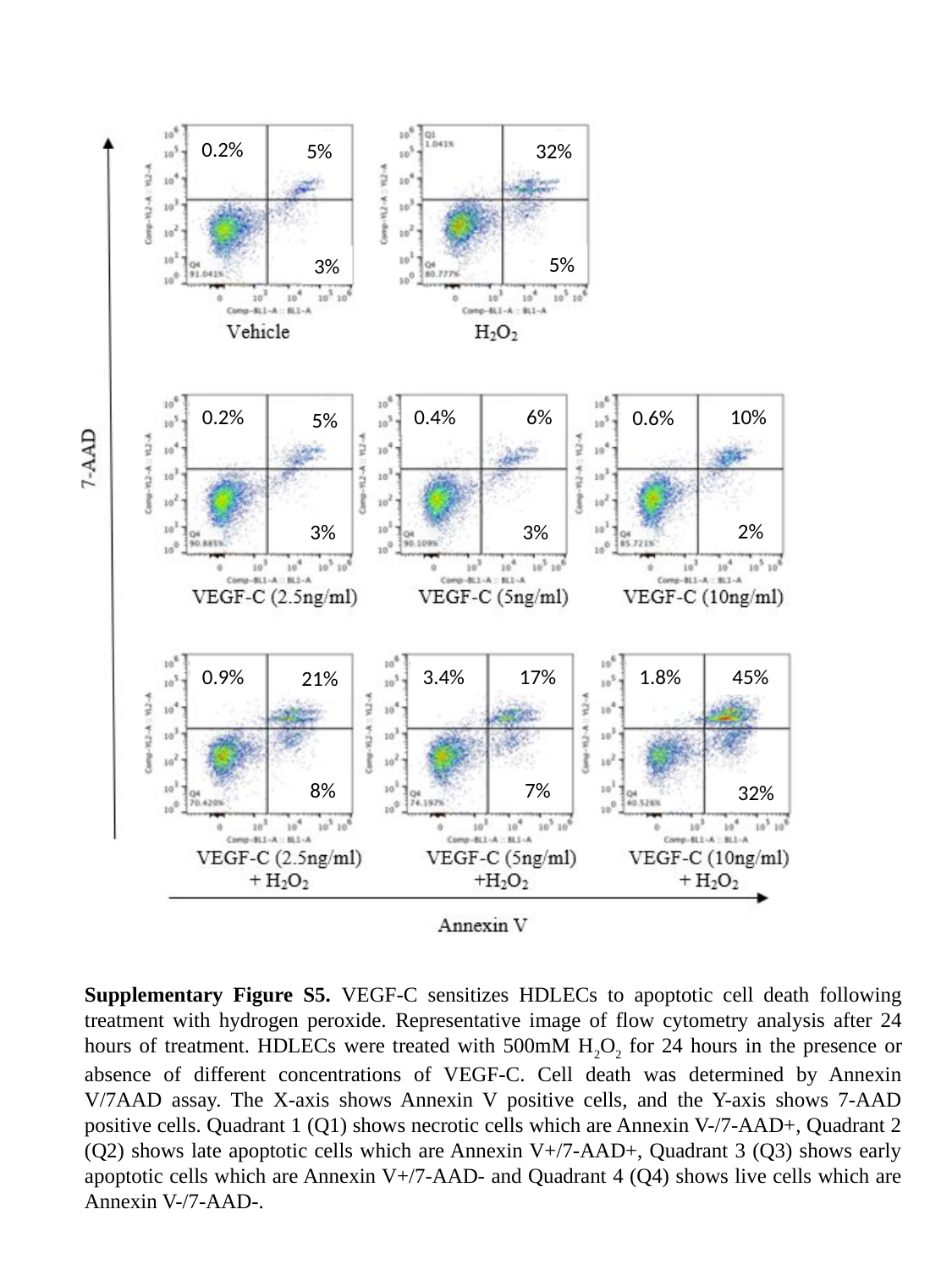

0.2%
5%
32%
5%
3%
0.2%
6%
0.4%
10%
0.6%
5%
2%
3%
3%
0.9%
1.8%
45%
17%
3.4%
21%
8%
7%
32%
Supplementary Figure S5. VEGF-C sensitizes HDLECs to apoptotic cell death following treatment with hydrogen peroxide. Representative image of flow cytometry analysis after 24 hours of treatment. HDLECs were treated with 500mM H2O2 for 24 hours in the presence or absence of different concentrations of VEGF-C. Cell death was determined by Annexin V/7AAD assay. The X-axis shows Annexin V positive cells, and the Y-axis shows 7-AAD positive cells. Quadrant 1 (Q1) shows necrotic cells which are Annexin V-/7-AAD+, Quadrant 2 (Q2) shows late apoptotic cells which are Annexin V+/7-AAD+, Quadrant 3 (Q3) shows early apoptotic cells which are Annexin V+/7-AAD- and Quadrant 4 (Q4) shows live cells which are Annexin V-/7-AAD-.

## Slide 6
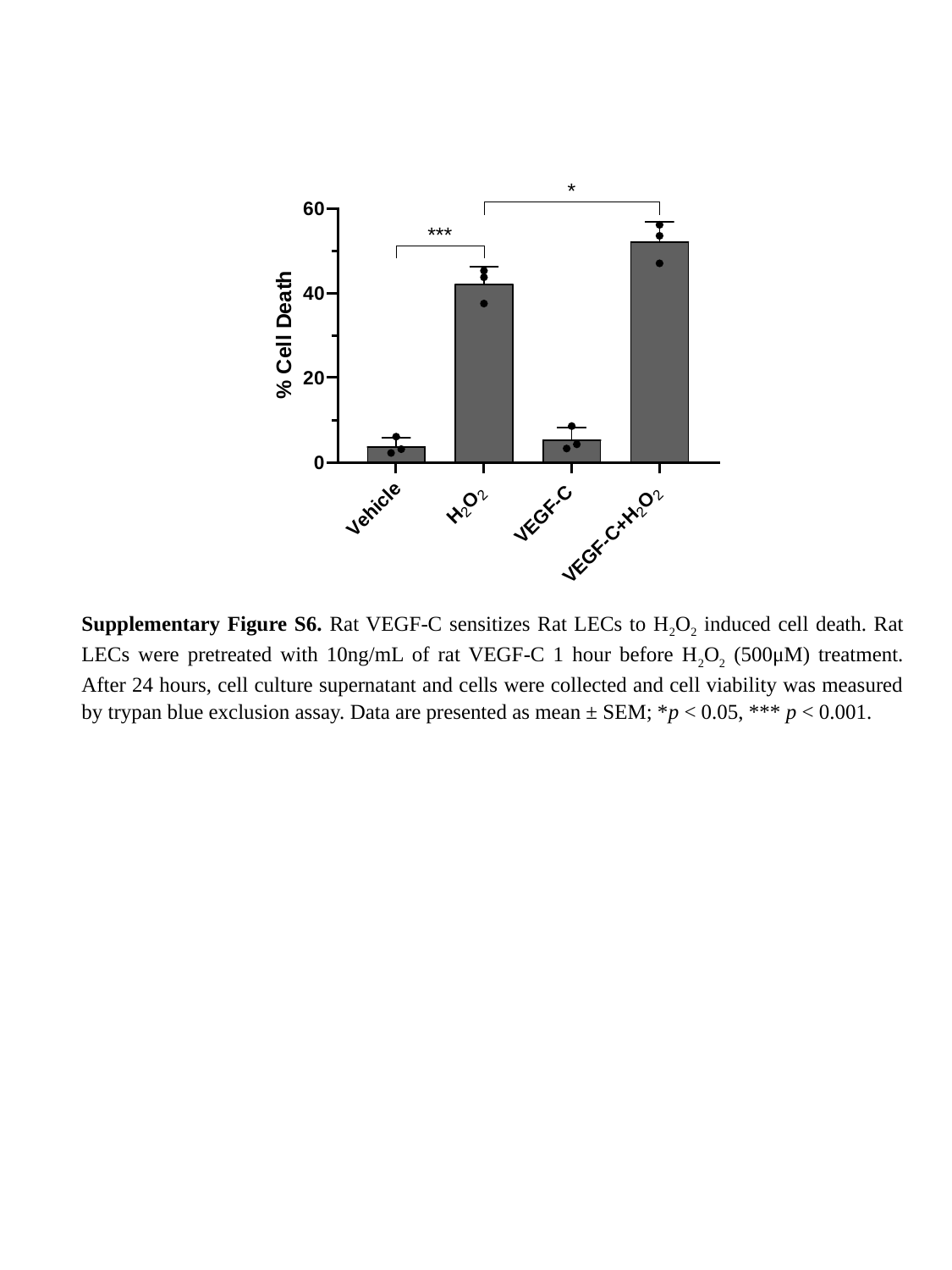

Supplementary Figure S6. Rat VEGF-C sensitizes Rat LECs to H2O2 induced cell death. Rat LECs were pretreated with 10ng/mL of rat VEGF-C 1 hour before H2O2 (500μM) treatment. After 24 hours, cell culture supernatant and cells were collected and cell viability was measured by trypan blue exclusion assay. Data are presented as mean ± SEM; *p < 0.05, *** p < 0.001.

## Slide 7
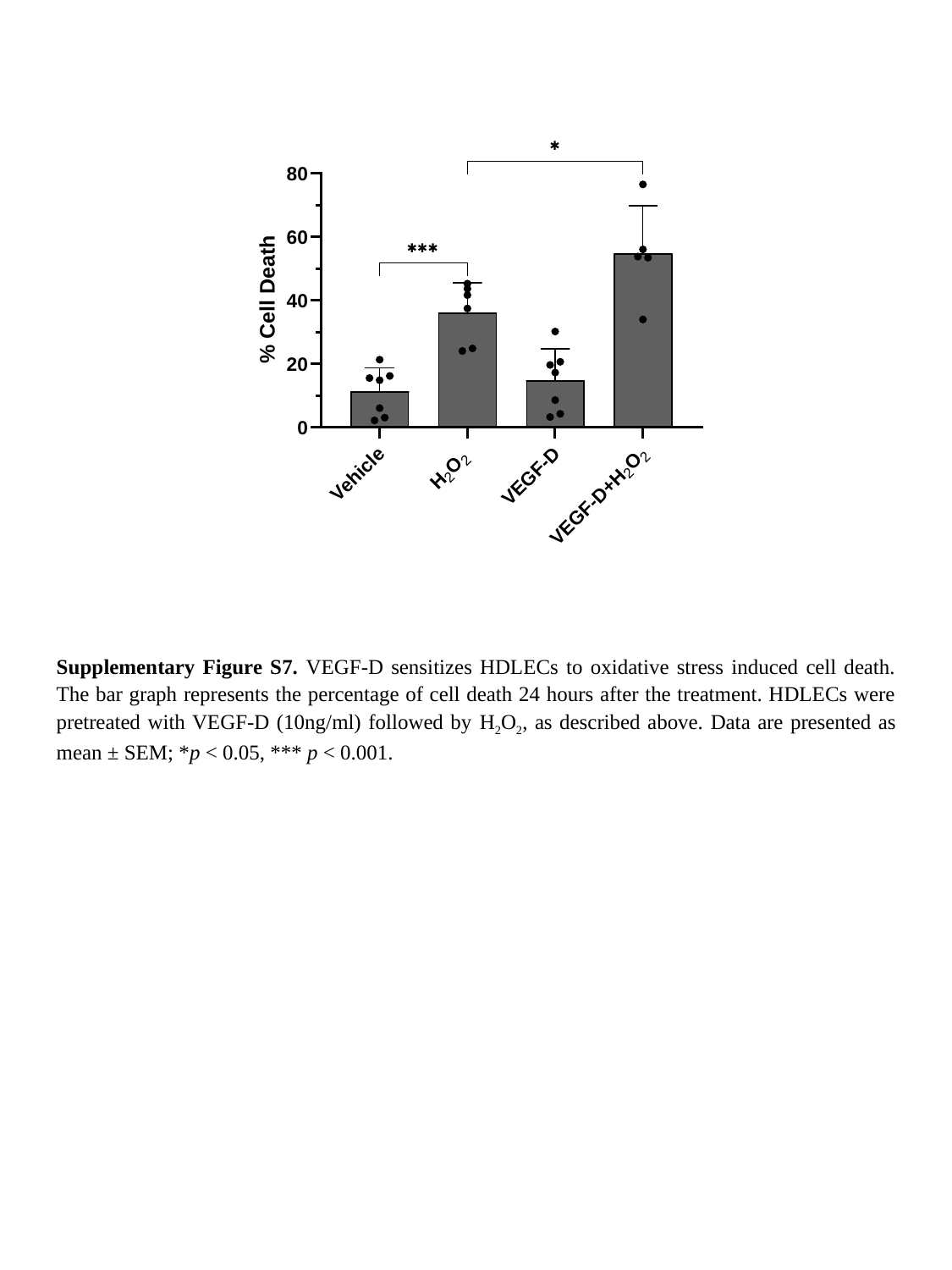

Supplementary Figure S7. VEGF-D sensitizes HDLECs to oxidative stress induced cell death. The bar graph represents the percentage of cell death 24 hours after the treatment. HDLECs were pretreated with VEGF-D (10ng/ml) followed by H2O2, as described above. Data are presented as mean ± SEM; *p < 0.05, *** p < 0.001.

## Slide 8
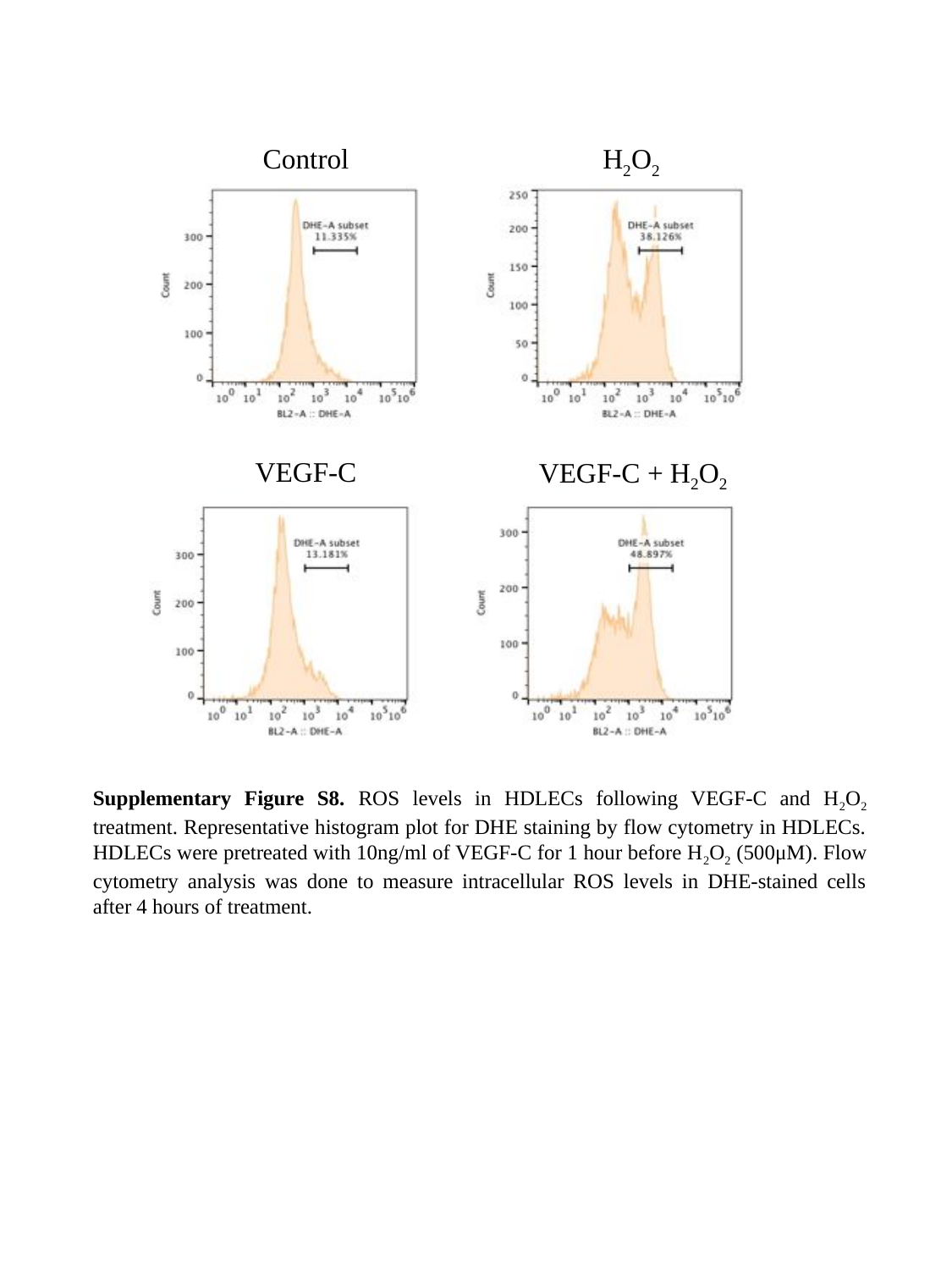

Control
H2O2
VEGF-C
VEGF-C + H2O2
Supplementary Figure S8. ROS levels in HDLECs following VEGF-C and H2O2 treatment. Representative histogram plot for DHE staining by flow cytometry in HDLECs. HDLECs were pretreated with 10ng/ml of VEGF-C for 1 hour before H2O2 (500μM). Flow cytometry analysis was done to measure intracellular ROS levels in DHE-stained cells after 4 hours of treatment.

## Slide 9
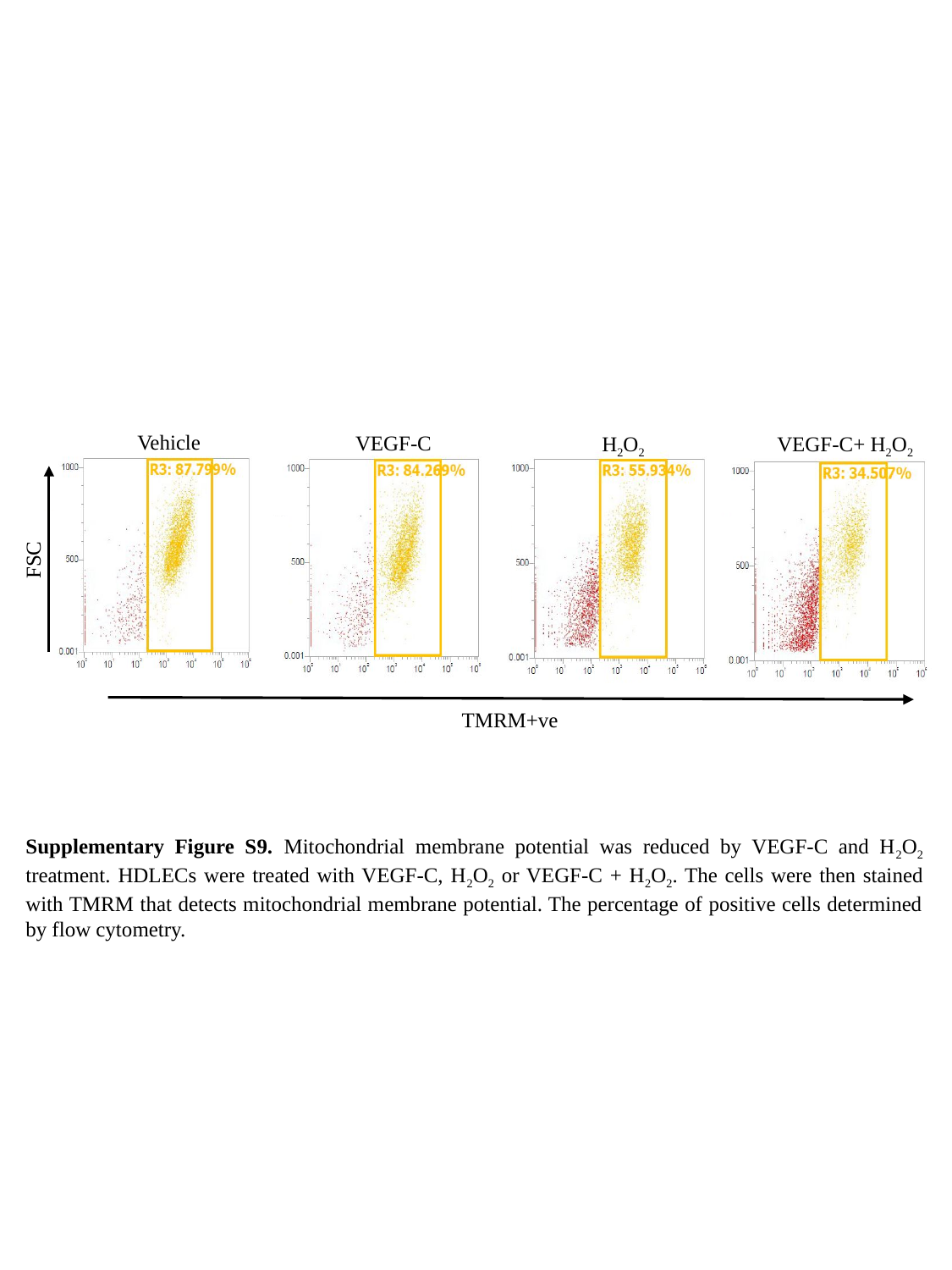

Vehicle
VEGF-C
VEGF-C+ H2O2
H2O2
FSC
TMRM+ve
Supplementary Figure S9. Mitochondrial membrane potential was reduced by VEGF-C and H2O2 treatment. HDLECs were treated with VEGF-C, H2O2 or VEGF-C + H2O2. The cells were then stained with TMRM that detects mitochondrial membrane potential. The percentage of positive cells determined by flow cytometry.

## Slide 10
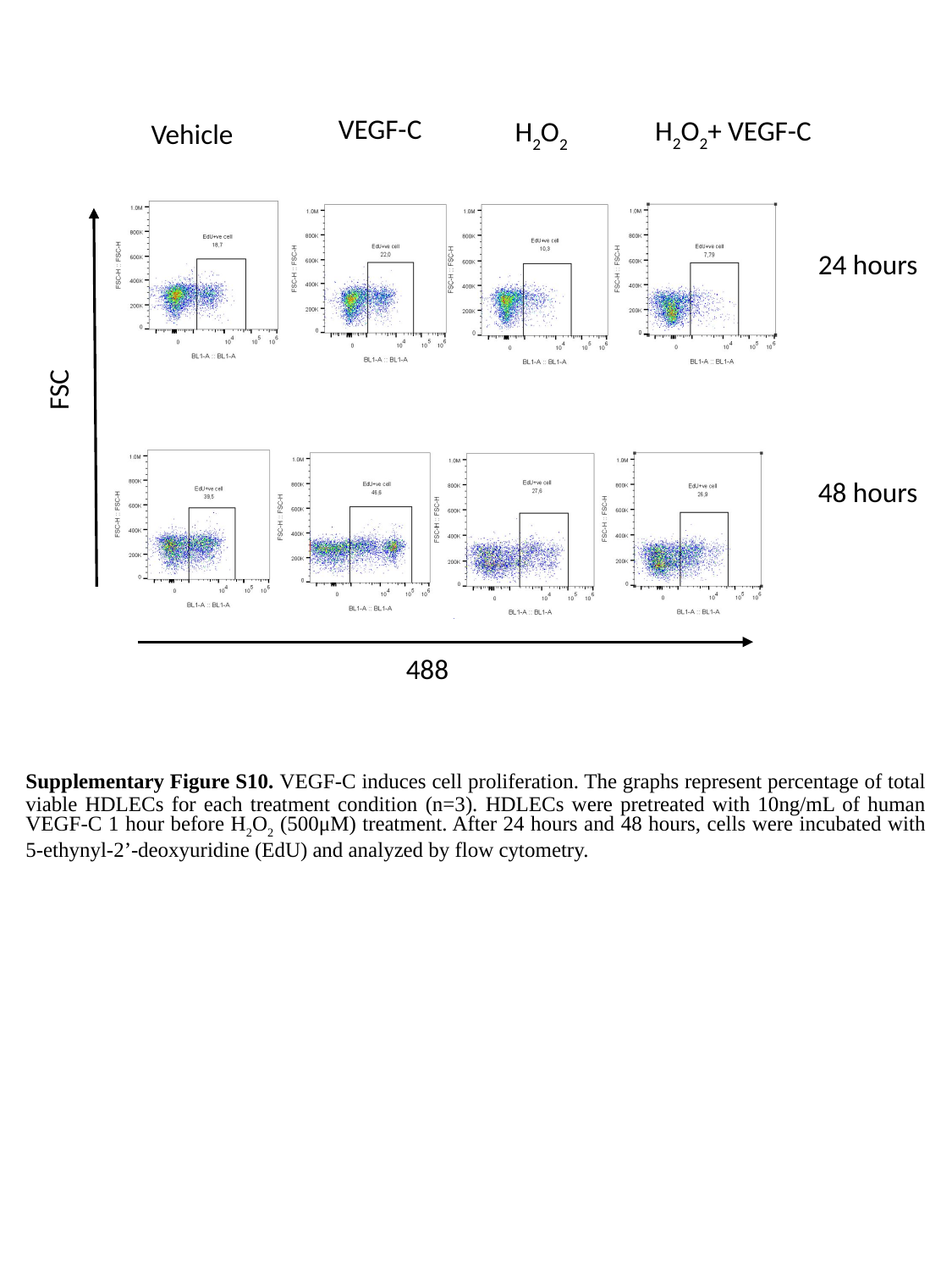

VEGF-C
H2O2+ VEGF-C
H2O2
Vehicle
24 hours
FSC
48 hours
488
Supplementary Figure S10. VEGF-C induces cell proliferation. The graphs represent percentage of total viable HDLECs for each treatment condition (n=3). HDLECs were pretreated with 10ng/mL of human VEGF-C 1 hour before H2O2 (500μM) treatment. After 24 hours and 48 hours, cells were incubated with 5-ethynyl-2’-deoxyuridine (EdU) and analyzed by flow cytometry.

## Slide 11
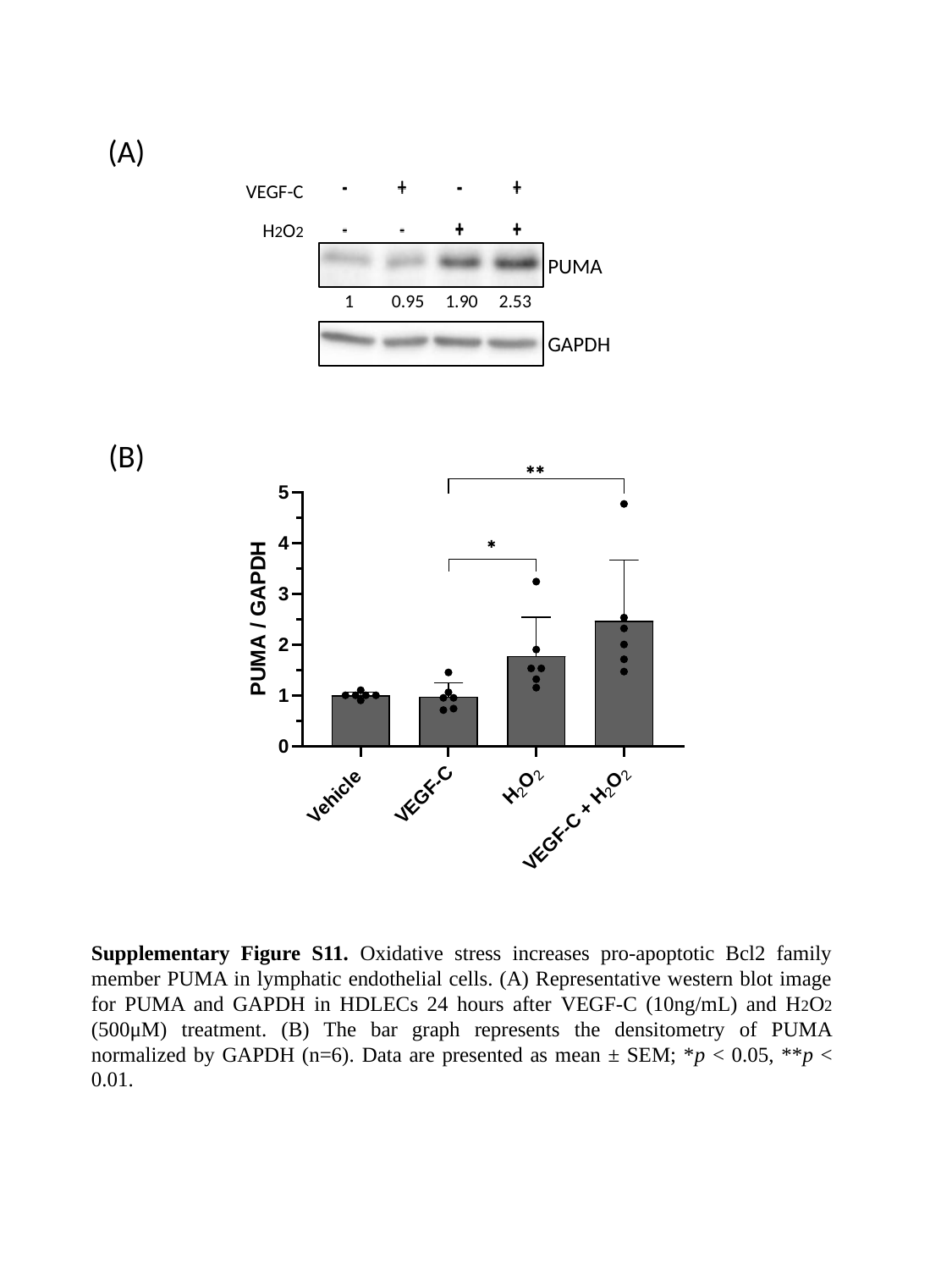

(A)
VEGF-C
H2O2
PUMA
 1 0.95 1.90 2.53
GAPDH
(B)
Supplementary Figure S11. Oxidative stress increases pro-apoptotic Bcl2 family member PUMA in lymphatic endothelial cells. (A) Representative western blot image for PUMA and GAPDH in HDLECs 24 hours after VEGF-C (10ng/mL) and H2O2 (500μM) treatment. (B) The bar graph represents the densitometry of PUMA normalized by GAPDH (n=6). Data are presented as mean ± SEM; *p < 0.05, **p < 0.01.

## Slide 12
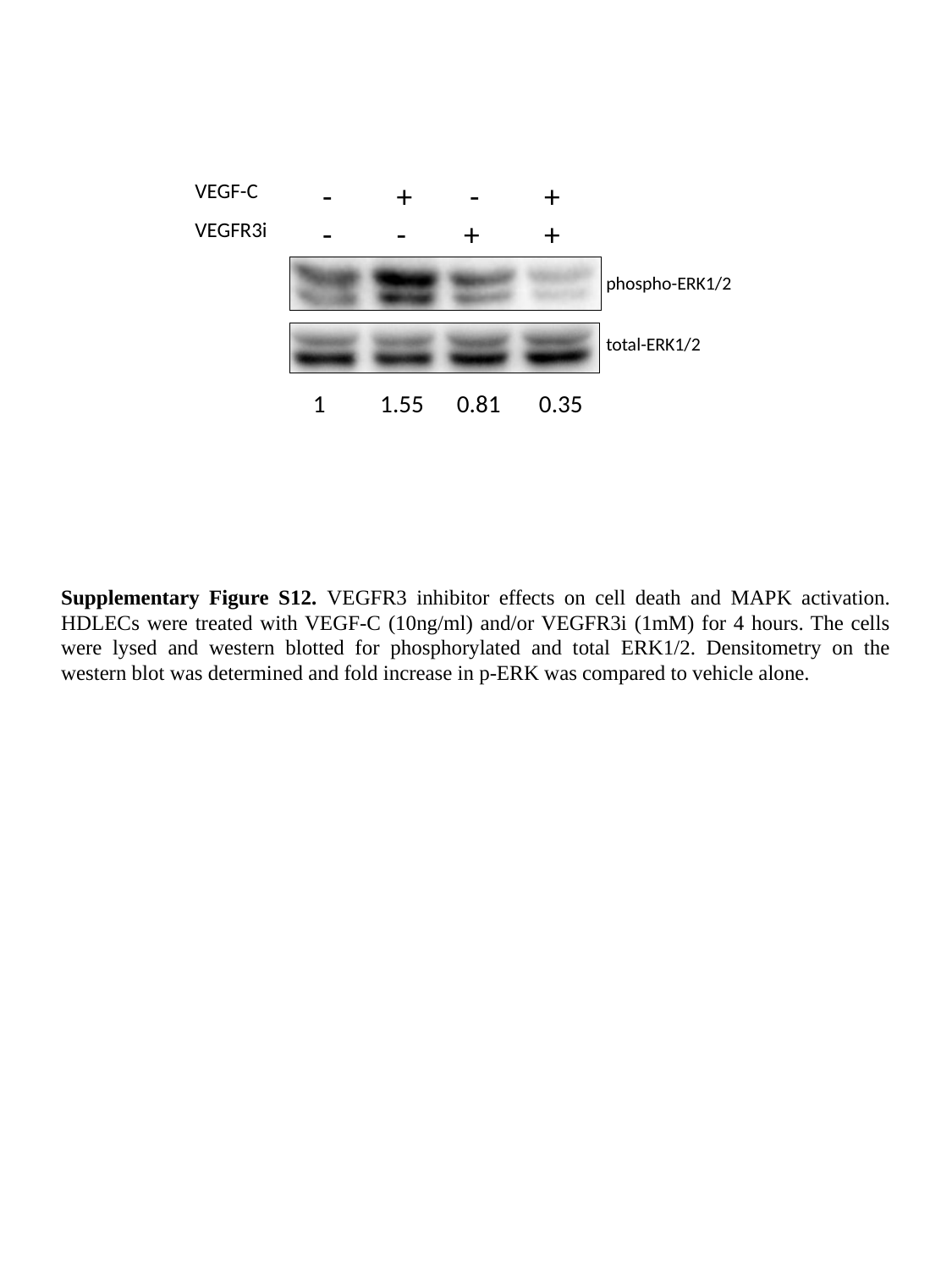

- + - +
 - - + +
VEGF-C
VEGFR3i
phospho-ERK1/2
total-ERK1/2
 1 1.55 0.81 0.35
Supplementary Figure S12. VEGFR3 inhibitor effects on cell death and MAPK activation. HDLECs were treated with VEGF-C (10ng/ml) and/or VEGFR3i (1mM) for 4 hours. The cells were lysed and western blotted for phosphorylated and total ERK1/2. Densitometry on the western blot was determined and fold increase in p-ERK was compared to vehicle alone.

## Slide 13
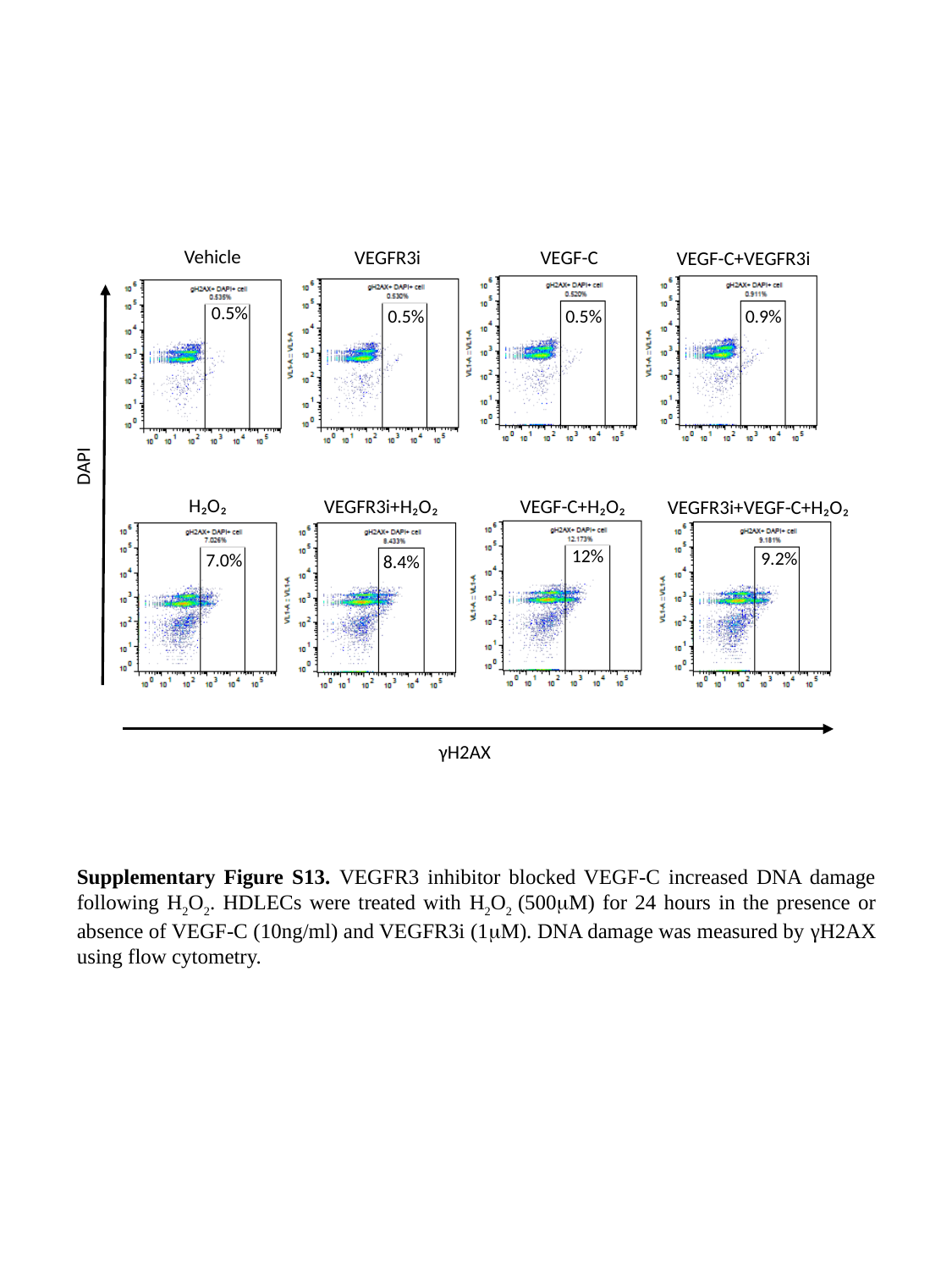

Vehicle
VEGFR3i
VEGF-C
VEGF-C+VEGFR3i
DAPI
H₂O₂
VEGFR3i+H₂O₂
VEGF-C+H₂O₂
VEGFR3i+VEGF-C+H₂O₂
γH2AX
0.5%
0.5%
0.5%
0.9%
12%
9.2%
7.0%
8.4%
Supplementary Figure S13. VEGFR3 inhibitor blocked VEGF-C increased DNA damage following H2O2. HDLECs were treated with H2O2 (500mM) for 24 hours in the presence or absence of VEGF-C (10ng/ml) and VEGFR3i (1mM). DNA damage was measured by γH2AX using flow cytometry.

## Slide 14
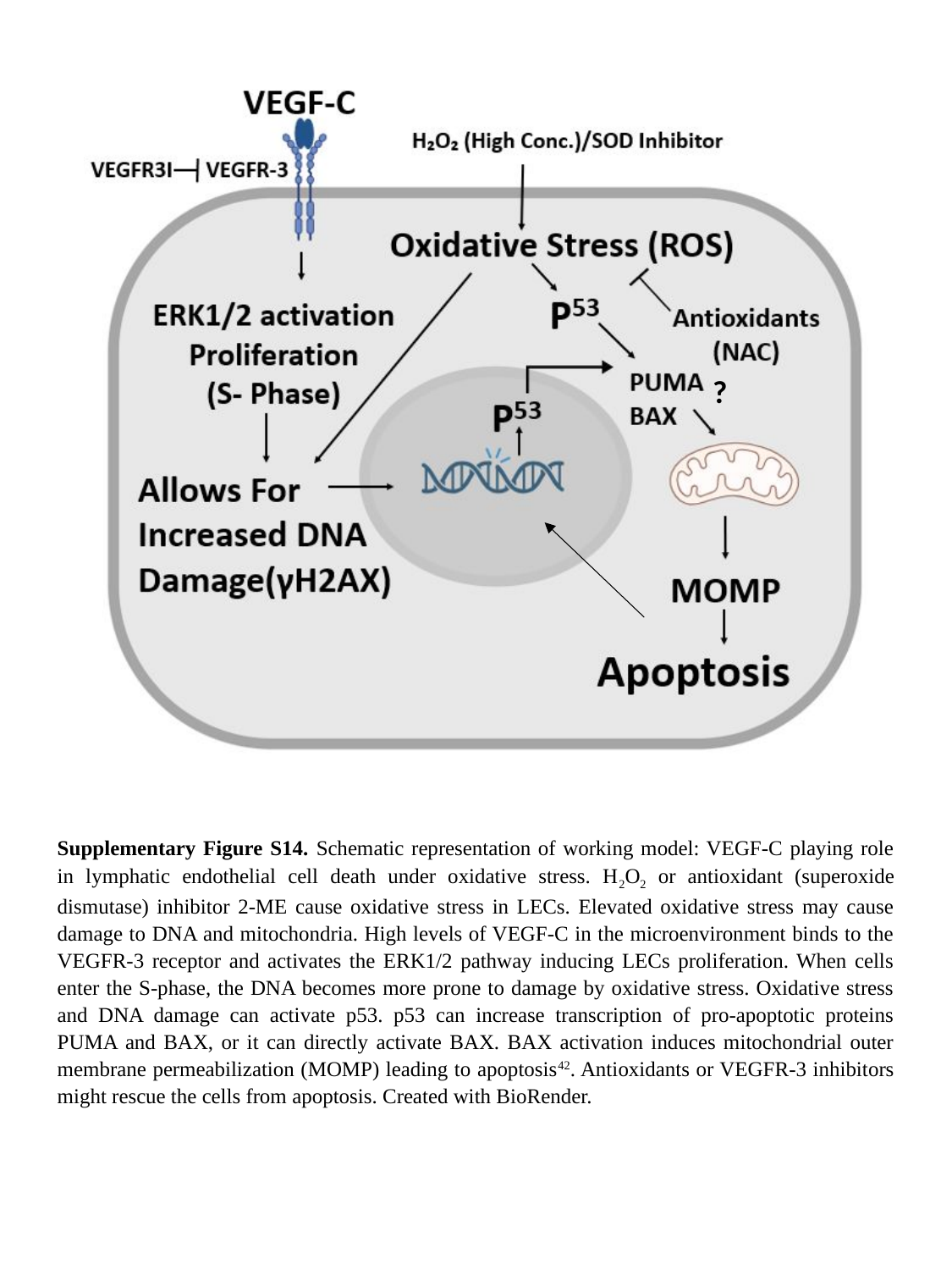

?
Supplementary Figure S14. Schematic representation of working model: VEGF-C playing role in lymphatic endothelial cell death under oxidative stress. H2O2 or antioxidant (superoxide dismutase) inhibitor 2-ME cause oxidative stress in LECs. Elevated oxidative stress may cause damage to DNA and mitochondria. High levels of VEGF-C in the microenvironment binds to the VEGFR-3 receptor and activates the ERK1/2 pathway inducing LECs proliferation. When cells enter the S-phase, the DNA becomes more prone to damage by oxidative stress. Oxidative stress and DNA damage can activate p53. p53 can increase transcription of pro-apoptotic proteins PUMA and BAX, or it can directly activate BAX. BAX activation induces mitochondrial outer membrane permeabilization (MOMP) leading to apoptosis42. Antioxidants or VEGFR-3 inhibitors might rescue the cells from apoptosis. Created with BioRender.
